# Supplementary material for: Human aminolevulinate synthase structure reveals a eukaryotic-specific autoinhibitory loop regulating substrate binding and product release
Source: Nat Commun. 2020 Jun 4;11:2813. doi: 10.1038/s41467-020-16586-x (PMC7272653; doi:10.1038/s41467-020-16586-x)
Supplement: Supplementary file 1 — Supplementary Information [file 41467_2020_16586_MOESM1_ESM.docx]

# Human aminolevulinate synthase structure reveals a eukaryotic-specific autoinhibitory loop regulating substrate binding and product release

Henry J. Bailey^1,5^, Gustavo A. Bezerra^1,5^, Jason R. Marcero^2,5^, Siladitya Padhi^3,5^, William R. Foster^1^, Elzbieta Rembeza^1^, Arijit Roy^3^, David F. Bishop^4^, Robert J. Desnick^4^, Gopalakrishnan Bulusu^3^, Harry A. Dailey, Jr.^2^, Wyatt W. Yue^1*^

^1^Structural Genomics Consortium, Nuffield Department of Medicine, University of Oxford, UK OX3 7DQ

^2^Department of Biochemistry and Molecular Biology, University of Georgia, Athens GA 30602

^3^TCS Innovation Labs-Hyderabad (Life Sciences Division), Tata Consultancy Services Ltd, Hyderabad 500081, India

^4^Department of Genetics and Genomics Sciences, Icahn School of Medicine at Mount Sinai, New York, NY 10029 USA

^5^These authors contributed equally

*Corresponding author: Wyatt W. Yue, [wyatt.yue@sgc.ox.ac.uk](mailto:wyatt.yue@sgc.ox.ac.uk), +44 (0)1865 617757

Supplementary Information

Supplementary Tables 1-5

Supplementary Figs. 1-18

Supplementary References

**Supplementary Table 1 Data collection and sample parameters for SAXS experiements.**

| Sample | hsALAS2_ΔN142_ | hsALAS2_ΔN142ΔC545_ |
| --- | --- | --- |
| Structural Parameters |  |  |
| Guinier |  |  |
| I(0) (cm^3^) | 0.06 | 0.07 |
| Rg (Å) | 33.33 | 31.61 |
| P(r) |  |  |
| I(0) (cm^3^) | 0.06 | 0.07 |
| Rg (Å) | 33.37 | 31.64 |
| Dmax (Å) | 122 | 121 |
| Shape model-fitting results |  |  |
| DAMMIF | default parameters, 13 calculations | default parameters, 13 calculations |
| q range for fitting | 8.15E-03 - 2.46E-01 | 1.13E-02 - 2.57E-01 |
| Symmetry, anisotropy assumptions | P2, none | P2, none |
| χ^2^ range | 1.351 – 1.641 | 1.134 – 1.379 |

hsALAS2_ΔN142_ and hsALAS2_ΔN142ΔC545_ constructs are referred to in Supplementary Fig. 2.

**Supplementary Table 2 Missense mutations reported to cause XLSA**

| No. | Exon | DNA change | Amino acid change | # Unrelated pedigrees | Supplementary References |
| --- | --- | --- | --- | --- | --- |
| 1 | 5 | c.462G>A | p.Met154Ile | 1 | ^1^ |
| 2 | 5 | c.466A>G | p.Lys156Glu | 2 | ^2, 3^ |
| 3 | 5 | c.475G>A | p.Asp159Asn | 1 | ^4^ |
| 4 | 5 | c.475G>T | p.Asp159Tyr | 1 | ^5^ |
| 5 | 5 | c.481A>G | p.Thr161Ala | 1 | ^6^ |
| 6 | 5 | c.485A>G | p.Tyr162Cys | 1 | ^7^ |
| 7 | 5 | c.488G>A | p.Arg163His | 2 | ^8^ |
| 8 | 5 | c.495C>A | p.Phe165Leu | 2 | ^9, 10^ |
| 9 | 5 | c.508C>A | p.Arg170Ser | 2 | ^11, 12^ |
| 10 | 5 | c.508C>T | p.Arg170Cys | 6 | ^10, 13, 14^ |
| 11 | 5 | c.509G>T | p.Arg170Leu | 4 | ^15, 16^ |
| 12 | 5 | c.509G>A | p.Arg170His | 5 | ^7, 14, 15^ |
| 13 | 5 | c.514G>A | p.Ala172Thr | 2 | ^17^ |
| 14 | 5 | c.569A>T | p.Asp190Val | 1 | ^18^ |
| 15 | 5 | c.577G>T | p.Val193Phe | 1 | ^19^ |
| 16 | 5 | c.595T>C | p.Tyr199His | 1 | ^20^ |
| 17 | 5 | c.606G>A | p.Met202Ile | 4 | ^7, 19^ |
| 18 | 5 | c.611G>A | p.Arg204Gln | 2 | ^21, 22^ |
| 19 | 5 | c.622G>T | p.Val208Phe | 1 | ^23^ |
| 20 | 6 | c.653G>A | p.Arg218His | 1 | ^16^ |
| 21 | 6 | c.670G>C | p.Gly224Arg | 1 | Bishop-unpublished |
| 22 | 6 | c.679C>T | p.Arg227Cys | 3 | ^24, 25^ |
| 23 | 6 | c.724G>A | p.Glu242Lys | 2 | ^16, 25^ |
| 24 | 6 | c.751T>C | p.Ser251Pro | 1 | ^26^ |
| 25 | 6 | c.776T>G | p.Phe259Cys | 1 | ^19^ |
| 26 | 6 | c.787G>A | p.Asp263Asn | 3 | ^16, 27^ |
| 27 | 6 | c.791C>A | p.Ser264Tyr | 1 | ^28^ |
| 28 | 6 | c.800T>C | p.Phe267Ser | 1 | ^19^ |
| 29 | 7 | c.824G>A | p.Gly275Glu | 1 | ^29^ |
| 30 | 7 | c.828C>G | p.Cys276Trp | 1 | ^30^ |
| 31 | 7 | c.866T>C | p.Ile289Thr | 1 | ^31^ |
| 32 | 7 | c.871G>A | p.Gly291Ser | 2 | ^19, 32^ |
| 33 | 7 | c.895A>C | p.Lys299Gln | 1 | ^17^ |
| 34 | 7 | c.897G>C | p.Lys299Asn | 1 | ^19^ |
| 35 | 7 | c.902T>C | p.Val301Ala | 2 | ^10, 33^ |
| 36 | 7 | c.919C>A | p.Pro307Thr | 1 | ^19^ |
| 37 | 7 | c.946A>G | p.Lys316Glu | 1 | ^19^ |
| 38 | 7 | c.971T>C | p.Ile324Thr | 2 | ^7, 34^ |
| 39 | 7 | c.984G>? | p.Glu328Asp | 1 | ^34^ |
| 40 | 8 | c.1016C>T | p.Pro339Leu | 1 | ^16^ |
| 41 | 8 | c.1051G>A | p.Gly351Arg | 1 | ^35^ |
| 42 | 8 | c.1054G>A | p.Ala352Thr | 1 | Bishop-unpublished |
| 43 | 8 | c.1070A>T | p.Asp357Val | 1 | ^19^ |
| 44 | 8 | c.1094A>G | p.Tyr365Cys | 1 | ^36^ |
| 45 | 8 | c.1093T>C | p.Tyr365His | 1 | ^7^ |
| 46 | 8 | c.1102C>T | p.Arg368Trp | 1 | ^34^ |
| 47 | 8 | c.1123C>T | p.Arg375Cys | 1 | ^16^ |
| 48 | 8 | c.1163C>G | p.Thr388Ser | 1 | ^19^ |
| 49 | 8 | c.1162A>C | p.Thr388Pro | 1 | ^37^ |
| 50 | 9 | c.1184G>A | p.Cys395Tyr | 1 | ^38^ |
| 51 | 9 | c.1193G>A | p.Gly398Asp | 1 | ^30^ |
| 52 | 9 | c.1218G>T | p.Leu406Phe | 1 | ^39^ |
| 53 | 9 | c.1231C>T | p.Arg411Cys | 7 | ^10, 14, 16, 20, 40^ |
| 54 | 9 | c.1232G>A | p.Arg411His | 4 | ^16, 41^ |
| 55 | 9 | c.1247G>A | p.Gly416Asp | 1 | ^42^ |
| 56 | 9 | c.1253T>G | p.Ile418Ser | 1 | ^43^ |
| 57 | 9 | c.1276A>G | p.Met426Val | 1 | ^18^ |
| 58 | 9 | c.1306C>T | p.Arg436Trp | 1 | ^44^ |
| 59 | 9 | c.1315A>G | p.Lys439Glu | 1 | ^45^ |
| 60 | 9 | c.1343G>A | p.Arg448Gln | 5 | ^13, 20, 42, 46^ |
| 61 | 9 | c.1349A>T | p.His450Leu | 1 | ^19^ |
| 62 | 9 | c.1354C>A | p.Arg452Ser | 6 | ^42, 47, 48, 49^ |
| 63 | 9 | c.1354C>T | p.Arg452Gly | 1 | ^16^ |
| 64 | 9 | c.1354C>T | p.Arg452Cys | 15 | ^2, 13, 14, 16, 19, 20, 42, 50^ |
| 65 | 9 | c.1355G>A | p.Arg452His | 26 | ^2, 10, 13, 14, 42, 50, 51, 52, 53, 54^ |
| 66 | 9 | c.1373G>A | p.Arg458His | 1 | ^55^ |
| 67 | 9 | c.1412G>A | p.Cys471Tyr | 2 | ^53, 56^ |
| 68 | 9 | c.1427T>A | p.Ile476Asn | 1 | ^57^ |
| 69 | 9 | c.1436G>A | p.Arg479Gln | 1 | ^58^ |
| 70 | 10 | c.1499A>G | p.Tyr500Cys | 2 | ^22, 25^ |
| 71 | 10 | c.1508C>T | p.Ala503Val | 2 | ^59, 60^ |
| 72 | 10 | c.1522A>T | p.Thr508Ser | 1 | ^30^ |
| 73 | 10 | c.1549C>T | p.Arg517Cys | 1 | ^30^ |
| 74 | 10 | c.1549C>T | p.Arg517Gly | 1 | ^10^ |
| 75 | 10 | c.1559C>T | p.Pro520Leu | 5 | ^10, 16, 61^ |
| 76 | 10 | c.1570C>G | p.His524Asp | 2 | ^62^ |
| 77 | 11 | c.1634T>A | p.Leu545Gln | 1 | ^19^ |
| 78 | 11 | c.1676G>A | p.Arg559His | 1 | ^55^ |
| 79 | 11 | c.1679G>A | p.Arg560His | 1 | ^63^ |
| 80 | 11 | c.1685T>C | p.Val562Ala | 1 | ^14^ |
| 81 | 11 | c.1688A>T | p.His563Leu | 1 | ^19^ |
| 82 | 11 | c.1693G>A | p.Glu565Lys | 1 | ^19^ |
| 83 | 11 | c.1699A>G | p.Met567Val | 1 | ^64^ |
| 84 | 11 | c.1701G>C | p.Met567Ile | 1 | ^14^ |
| 85 | 11 | c.1702A>G | p.Ser568Gly | 4 | ^65^ |
| 86 | 11 | c.1715G>A | p.Arg572His | 2 | ^16^ |

**Supplementary Table 3 Fragment-bound hsALAS2 structures from crystallography-based screening**

| PDB ID | Fragment-bound structure | Resolution (Å) | Chemical structure |
| --- | --- | --- | --- |
| [5QQY](https://www.rcsb.org/structure/5QQY) | Structure of hsALAS2 bound with PLP and fragment ***1*** (F9000350): (1-(thiophen-3-ylmethyl)piperidin-4-ol) | 1.49 |  |
| [5QR1](https://www.rcsb.org/structure/5QR1) | Structure of hsALAS2 bound with PLP and fragment ***2*** (F9000534): *N*-(cyclobutylmethyl)-1,5-dimethyl-1*H*-pyrazole-4-carboxamide | 1.44 |  |
| [5QRA](https://www.rcsb.org/structure/5QRA) | Structure of hsALAS2 bound with PLP and fragment ***3*** (F9000413): (4-methylazepan-1-yl)(thiazol-4-yl)methanone | 1.73 |  |
| [5QRC](https://www.rcsb.org/structure/5QRC) | Structure of hsALAS2 bound with PLP and fragment ***4*** (FM010110): 3-cyclohexyl-1-morpholinopropan-1-one | 1.83 |  |
| [5QRD](https://www.rcsb.org/structure/5QRD) | Structure of hsALAS2 bound with PLP and fragment ***5*** (F9000430): 4-((2-(methylsulfonyl)-1*H*-imidazol-1-yl)methyl)thiazole | 1.76 |  |
| [5QQW](https://www.rcsb.org/structure/5QQW) | Structure of hsALAS2 bound with PLP and fragment ***6*** (XS179878): 2-methyl-N-(pyridin-4-yl)furan-3-carboxamide | 1.56 |  |
| [5QQX](https://www.rcsb.org/structure/5QQX) | Structure of hsALAS2 bound with PLP and fragment ***7*** (F9000532): N-(1-ethyl-1H-pyrazol-4-yl)cyclobutanecarboxamide | 1.51 |  |
| [5QRE](https://www.rcsb.org/structure/5QRE) | Structure of hsALAS2 bound with PLP and fragment ***8*** (Z117233350): 3-ethyl-5-methyl-N-(5-methylisoxazol-3-yl)isoxazole-4-carboxamide | 1.68 |  |
| [5QQU](https://www.rcsb.org/structure/5QQU) | Structure of hsALAS2 bound with PLP and fragment ***9*** (F9000370): 5-(1,4-oxazepan-4-yl)picolinonitrile | 1.55 |  |

Data collection and refinement statistics can be found in Supplementary Data 1.

**Supplementary Table 4 Relative enzyme activities of recombinant hsALAS2 WT (0.025 μM) in the presence of small-molecule fragments 1 and 8.**

| Additive | Concentration | WT Activity | % WT Activity |
| --- | --- | --- | --- |
| DMSO | ‒ | 1.00 ± 0.06 | 100 ± 6 |
| Fragment **1** | 1 mM  5 mM | 0.84 ± 0.03  0.72 ± 0.02 | 84 ± 3  72 ± 2 |
| Fragment **8** | 1 mM  5 mM | 0.85 ± 0.04  0.78 ± 0.03 | 85 ± 4  78 ± 3 |

Activity values represent the mean of three biological replicates ± SD (each biological replicate is the average of two technical replicates) and have been normalized to WT enzyme treated with DMSO (diluent). Source data are provided as a Source Data file.

**Supplementary Table 5 Simulation box details**

| System | Number of atoms (including water) | Box dimensions (in Å) |
| --- | --- | --- |
| No PLP | 168,426 | 119.1 × 119.1 × 119.1 |
| With PLP | 168,478 | 119.1 × 119.1 × 119.1 |
| With substrates | 170,469 | 119.6 × 119.6 × 119.6 |


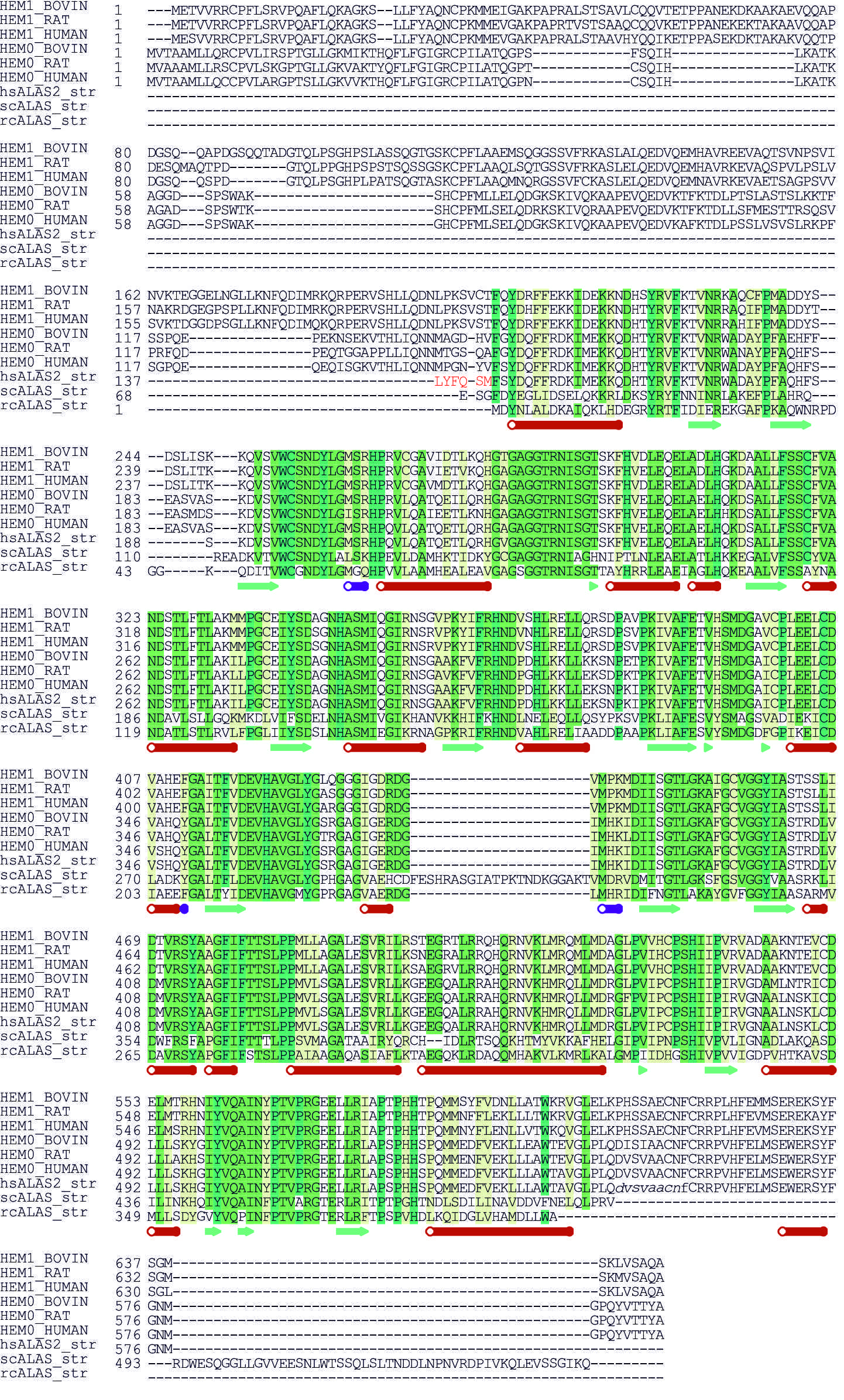


**Supplementary Fig. 1 Structure-based sequence alignment of ALAS orthologues.** Sequences of the crystallized constructs used in the structure determination of hsALAS2 (hsALAS2_str), *S. cerevisiae* ALAS (scALAS_str), and *R. capsulatus* ALAS (rcALAS_str) are aligned, on the basis of Cα-superposition of their structures. Additionally, full-length sequences of ALAS1 from bovine (HEM1_BOVIN), rat (HEM1_RAT) and human (HEM1_HUMAN), as well as full-length sequences of ALAS2 from bovine (HEM0_BOVIN), rat (HEM0_RAT) and human (HEM0_HUMAN) are appended to the alignment. For the hsALAS2_str sequence, vector-encoded residues at the beginning of the construct are denoted in red letters, and secondary structure assignment is shown at the bottom of the alignment (α-helix, red; 3_10_ helix, purple; β-strand, green).


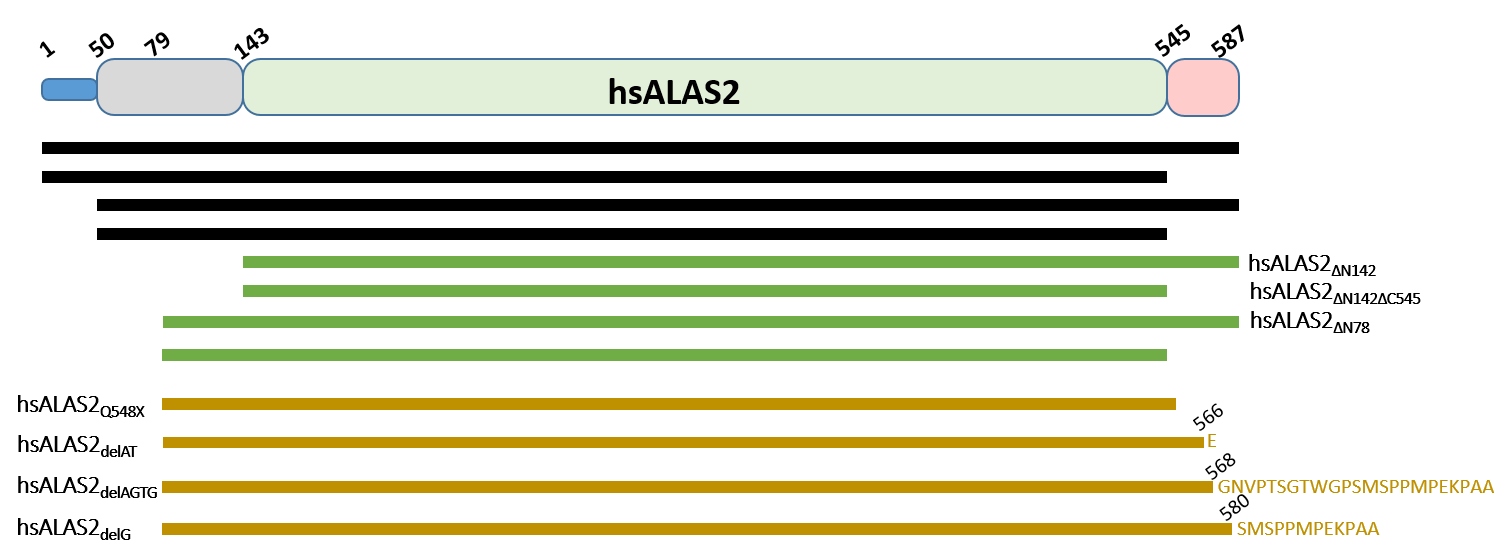


**Supplementary Fig. 2 Domain architecture of hsALAS2.** hsALAS2 is a 587-aa polypeptide (top) containing a mitochondrial targeting sequence (blue), an unstructured domain of unknown function (grey), a conserved catalytic domain (light green) and a C-terminal extension unique to eukaryotes (pink). Bottom*:* Black bars denote attempted constructs that were insoluble during expression tests, green bars represent soluble wild-type constructs in this study, and orange bars indicate constructs of disease-associated variants in this study.

**
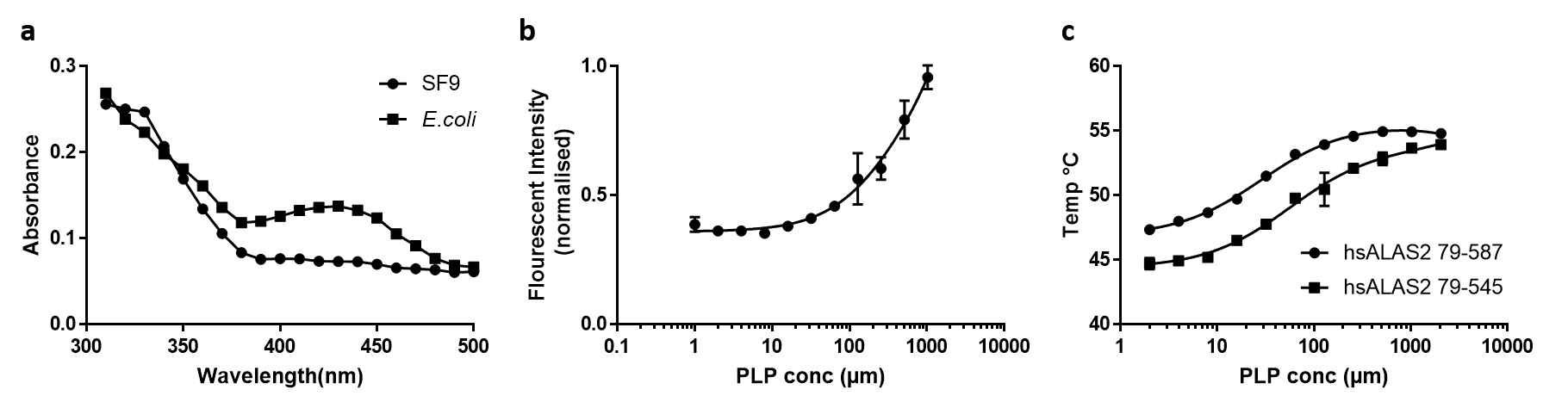
**

**Supplementary Fig. 3 PLP loading of recombinant hsALAS2.** (**a**) UV-Vis spectrum of hsALAS2_ΔN142_ comparing different ratios of the PLP tautomeric states of holoenzyme when isolated from insect cell (Sf9) or *E. coli* cell expression systems. *E.coli*-expressed hsALAS2 produces holoenzyme in the protonated ketoenamine tautomer (shown by absorption maximum at 420 nm), whereas insect cell-expressed hsALAS2 likely yields holoenzyme in the deprotonated enolimine tautomer (shown by absorption maximum at 335 nm and lack of absorbance at 420 nm). Raw data points plotted from one experiment. (**b**) A plot of fluorescence intensity increase with excitation at 440nm and emission at 520nm. Titration of excess PLP to insect cell-expressed hsALAS2_ΔN78_ shifts the equilibrium towards the protonated ketoenamine form, shown by increased fluorescence of the internal aldimine. Data are presented as mean values +/- SEM from three technical replicates (n=3). (**c**) Midpoint melting temperatures, determined by differential scanning fluorimetry, of insect cell-expressed hsALAS2_ΔN78_ and hsALAS2_ΔN78ΔC545_. The ketoenamine form resulting from titration of PLP to both proteins is thermally more stable. Data are presented as mean values +/- SEM, from n=3 technical replicates. Source data are provided as a Source Data file.


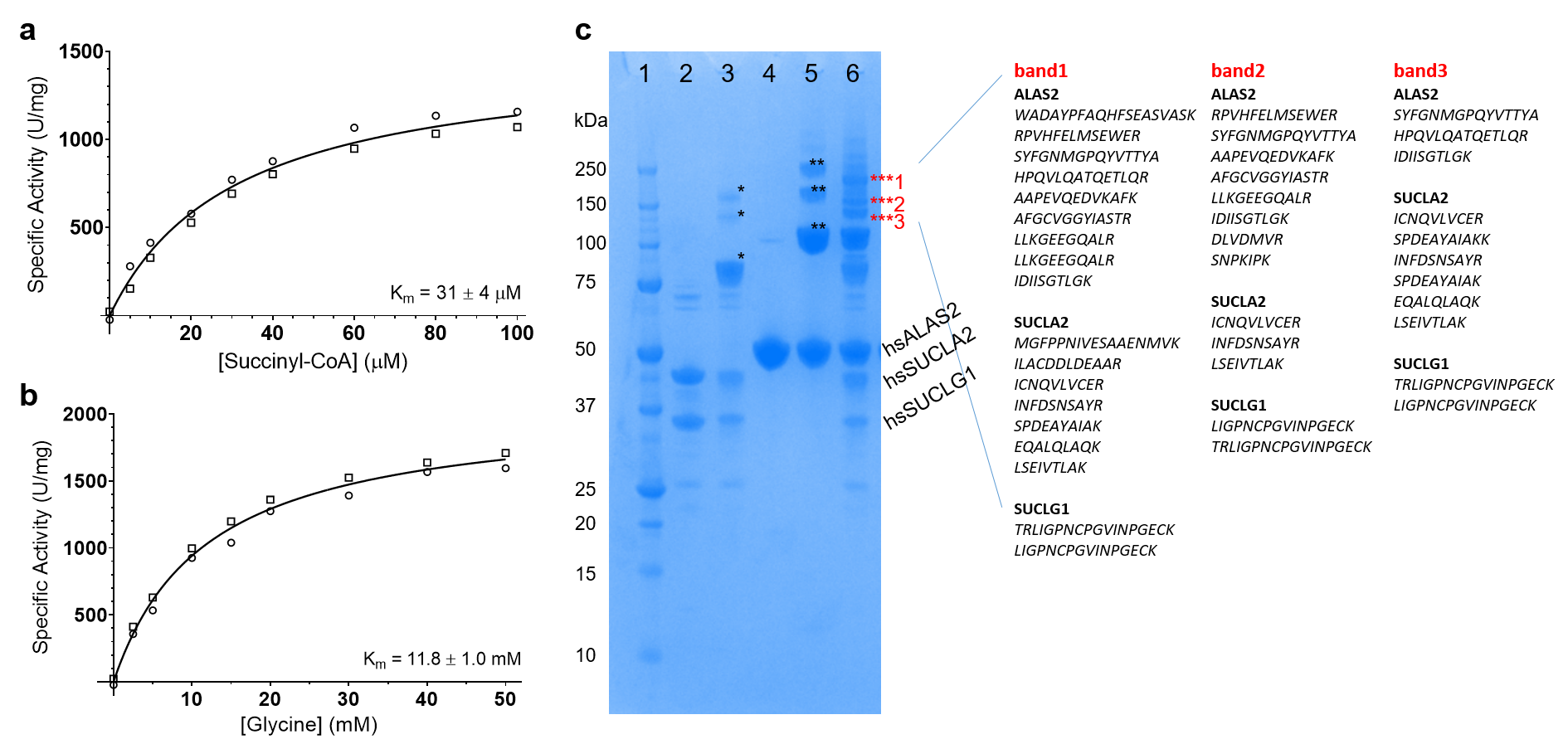


**Supplementary Fig. 4 Functional integrity of recombinant hsALAS2ΔN78 and hsALAS2_ΔN142_ proteins.** (**a, b**) Kinetic progress curves for recombinant hsALAS2_ΔN142_ protein overexpressed in Sf9 insect cells. Specific activity represents initial reaction velocity normalized to protein in each assay (4 µg mL^-1^), where succinyl-CoA and glycine were titrated in the presence of 50 mM glycine and 100 µM succinyl-CoA, respectively. One unit of activity (U) is the amount of enzyme needed to catalyze the formation of 1 nmol ALA per hour. Each regression line was fitted to data from two biological replicates (n=2), with the data for each replicate plotted as distinct symbols (circles and squares). (**c**) Crosslinking SDS-PAGE of interactions between hsALAS2ΔN78 and succinyl-CoA ligase. Results shown are representative of three independent experiments. Lane 1, molecular weight markers in kDa; Lane 2, purified succinyl-CoA ligase (SUCLG1-SUCLA2); Lane 3, SUCLG1-SUCLA2 after incubation with di(N-succinimidyl) glutarate crosslinker (DSG), showing crosslinked oligomeric species of SUCLG1-SUCLA2 (*); Lane 4, purified hsALAS2_ΔN78_; Lane 5, hsALAS2_ΔN78_ incubated with DSG, showing crosslinked oligomeric species of hsALAS2 (**); Lane 6, mixture of SUCLG1-SUCLA2 and hsALAS2ΔN78 incubated with DSG, showing crosslinked species representing the complex between SUCLG1-SUCLA2 and hsALAS2ΔN78 (***). Protein identity of all asterisked bands (1-3) were confirmed by tryptic digest MS/MS, in which mapped peptides corresponding to each of the three component proteins are shown in the inset figure. Source data are provided as a Source Data file.

**
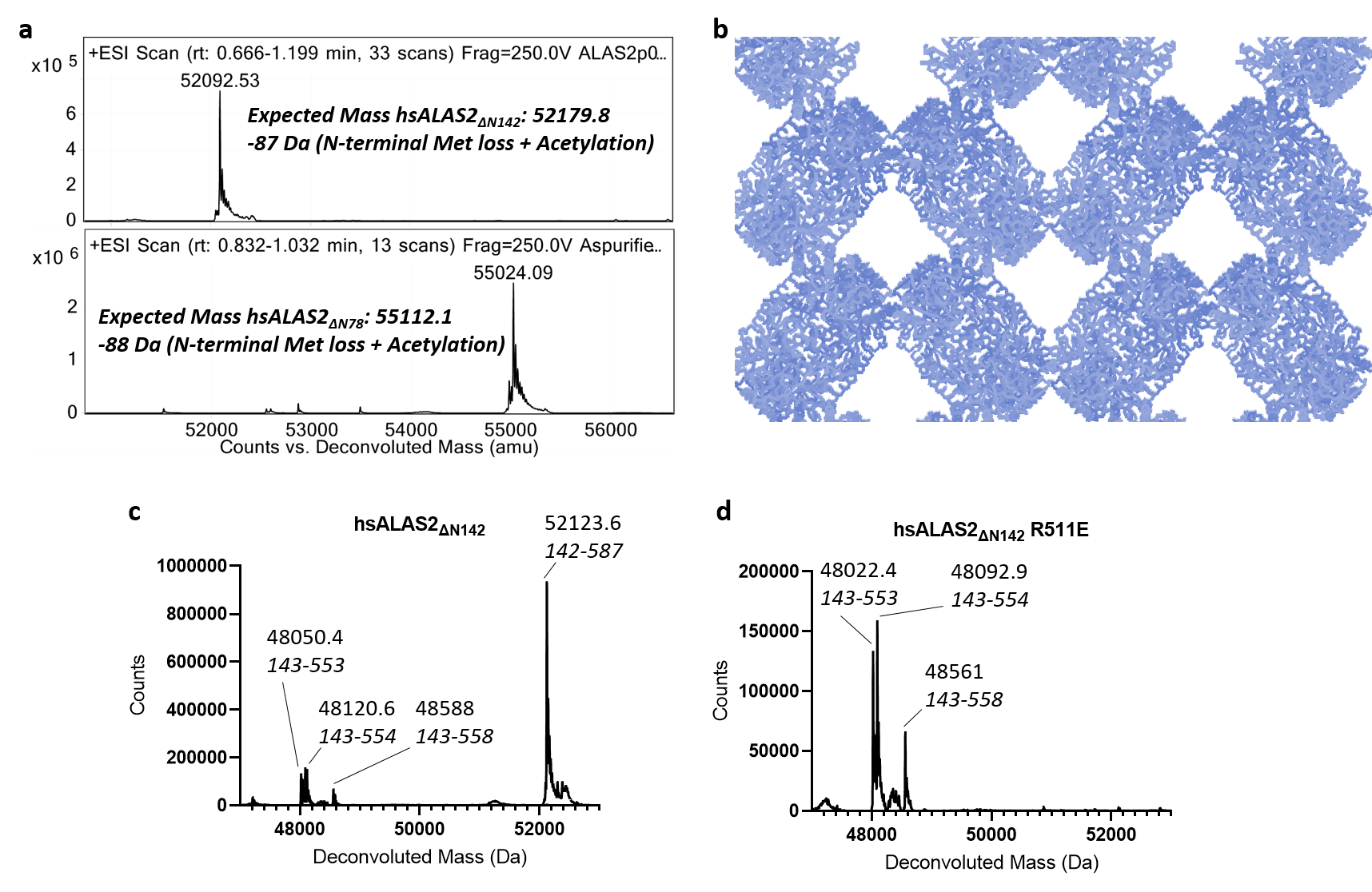
**

**Supplementary Fig. 5 N- and C-termini of our recombinant hsALAS2 proteins.** (**a**) Deconvoluted mass spectrum of both purified hsALAS2_ΔN142_ and hsALAS2_ΔN78_ proteins expressed in SF9 cells and used in structural studies are shown to have a fully intact N-terminus and C-terminus, after expression and purification. (**b**) Intermolecular packing of hsALAS2_ΔN78_ crystals, showing space between dimers (blue ribbons) that could account for the disordered N-terminus. (**c**) Deconvoluted mass spectrum of *E.coli* expressed hsALAS2ΔN142 shows predominantly intact protein (expected MW 52122.7 Da, measured MW 52123.6 Da) with three additional degradation peaks at low relative abundance to intact protein. Peak at 48588 Da corresponds to hsALAS2 143-558, peak at 48120.6 Da corresponds to hsALAS2 143-554 and peak at 48050.4 corresponds to hsALAS2 143-553 (**d**) Deconvoluted mass spectrum of *E.coli* expressed R511E variant of hsALAS2ΔN142 shows no signal for intact protein (expected MW 52095.49 Da) and high abundance of the three degradation peaks seen in WT. Peak at 48561 Da corresponds to R511E hsALAS2 143-558, peak at 48092.9 Da corresponds to R511E hsALAS2 143-554 and peak at 48022.4 corresponds to R511E hsALAS2 143-553. Source data for Supplementary Fig. 5a, c and d are provided as a Source Data file.


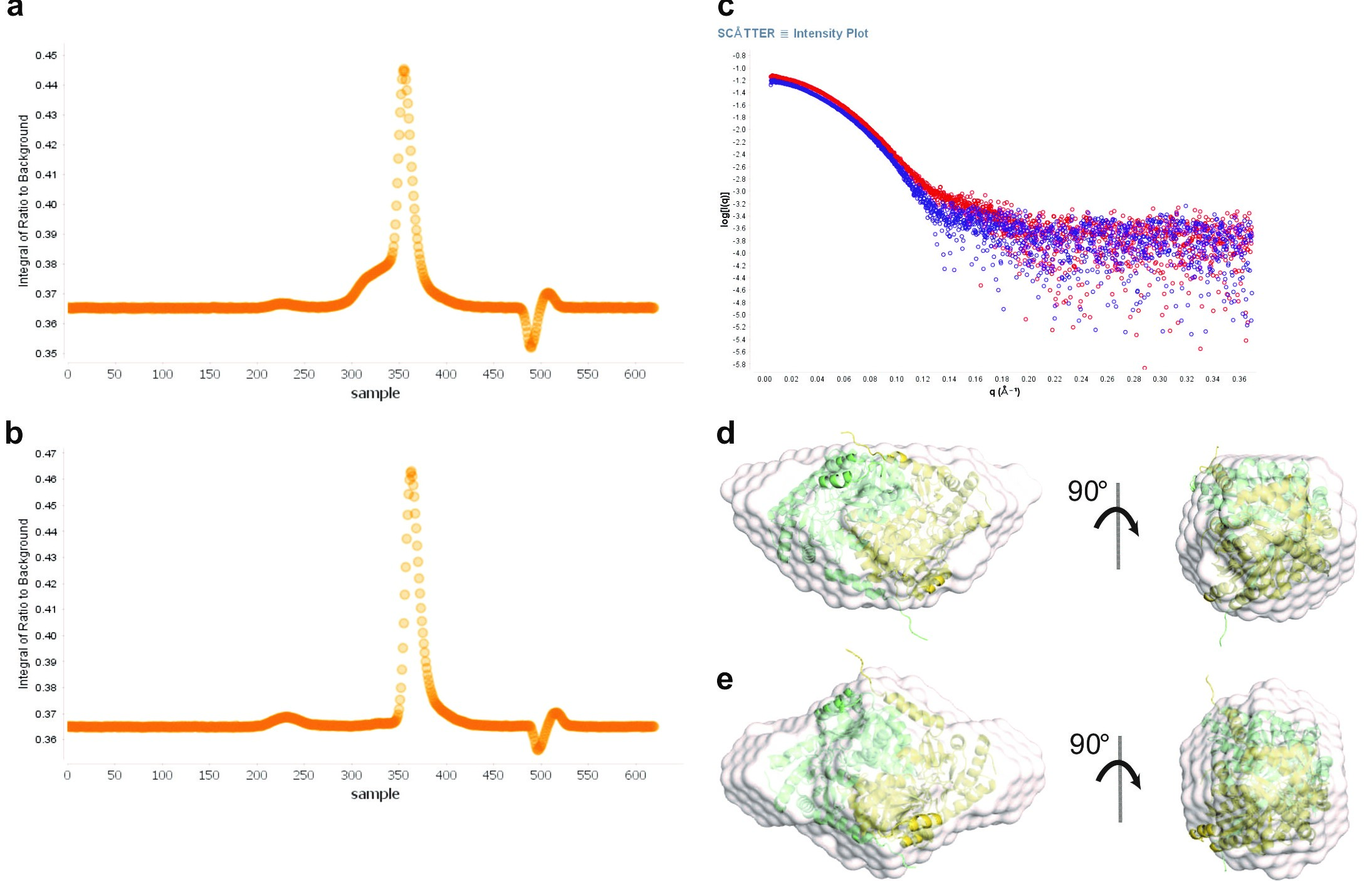


**Supplementary Fig. 6 Small angle X-ray scattering studies of hsALAS2_ΔN142_ and hsALAS2_ΔN142ΔC545_.** (**a**) SEC-SAXS signal plot of hsALAS2_ΔN142_, with each data point (orange circle) representing the integrated area of the ratio of the sample SAXS curve to the estimated background. (**b**) SEC-SAXS signal plot of hsALAS2_ΔN142ΔC545_, construct that contains only the catalytic domain without the C-terminal extension. (**c**) Log10 intensity plot of subtracted and merged SAXS frames. Red circles represent scattering data points from hsALAS2_ΔN142ΔC545_, and blue circles represent scattering data points from hsALAS2_ΔN142._ (**d**) Orthogonal views of the *ab initio* envelopes calculated from SAXS data for hsALAS2_ΔN142._ (**e**) Orthogonal views of the *ab initio* envelopes calculated from SAXS data for hsALAS2_ΔN142ΔC545._ Source data are provided as a Source Data file.


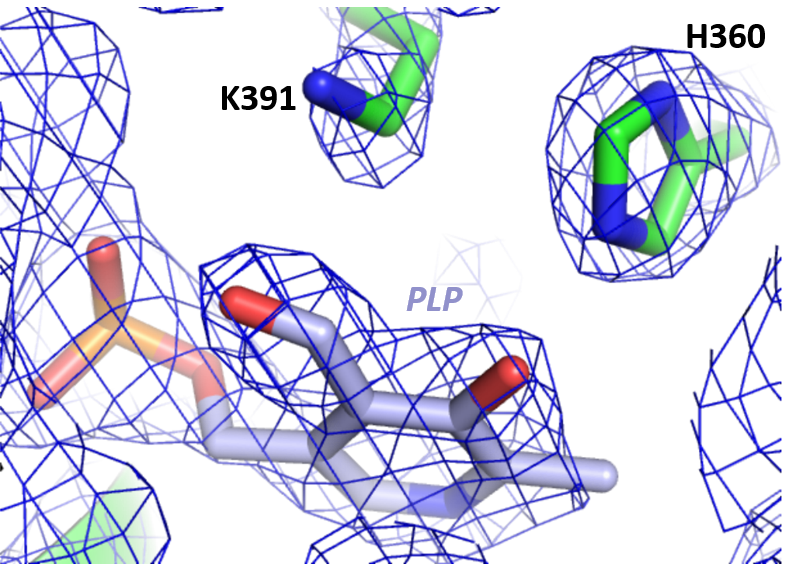


**Supplementary Fig. 7 PLP binding site.** 2*F*o − *F*c electron density map of the active site region, contoured at 1σ, revealing residues of hsALAS2 that interact with PLP. As expected, no covalent linkage was observed between PLP and its Schiff base Lys391 in the crystal.

**
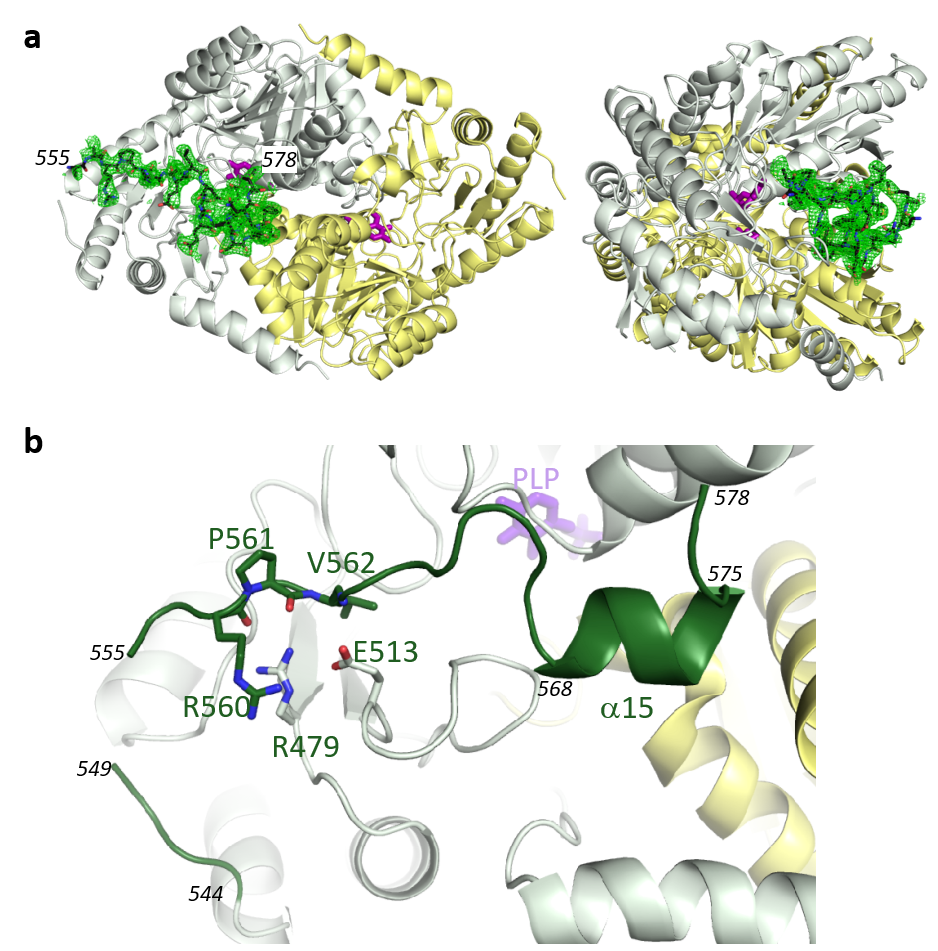
**

**Supplementary Fig. 8 The hsALAS2 C-terminal extension.** (**a**) Composite 2mFo-DFc omit map (by Phenix software) of the hsALAS2 Ct-extension, contoured at 1σ , shown for one subunit of the homodimer. Black lines represent Ct-extension residues 555-578 built onto the density. (**b**) Topology of an hsALAS2 Ct-extension (dark green), packing against the catalytic core of its own protomer (light green) and of the opposing protomer in the homodimer (yellow). PLP is shown as purple sticks. Interacting residues of the catalytic core and the unstructured portion of the Ct-extension are displayed. Residues 550-554 are disordered in the structure and hence not modeled.


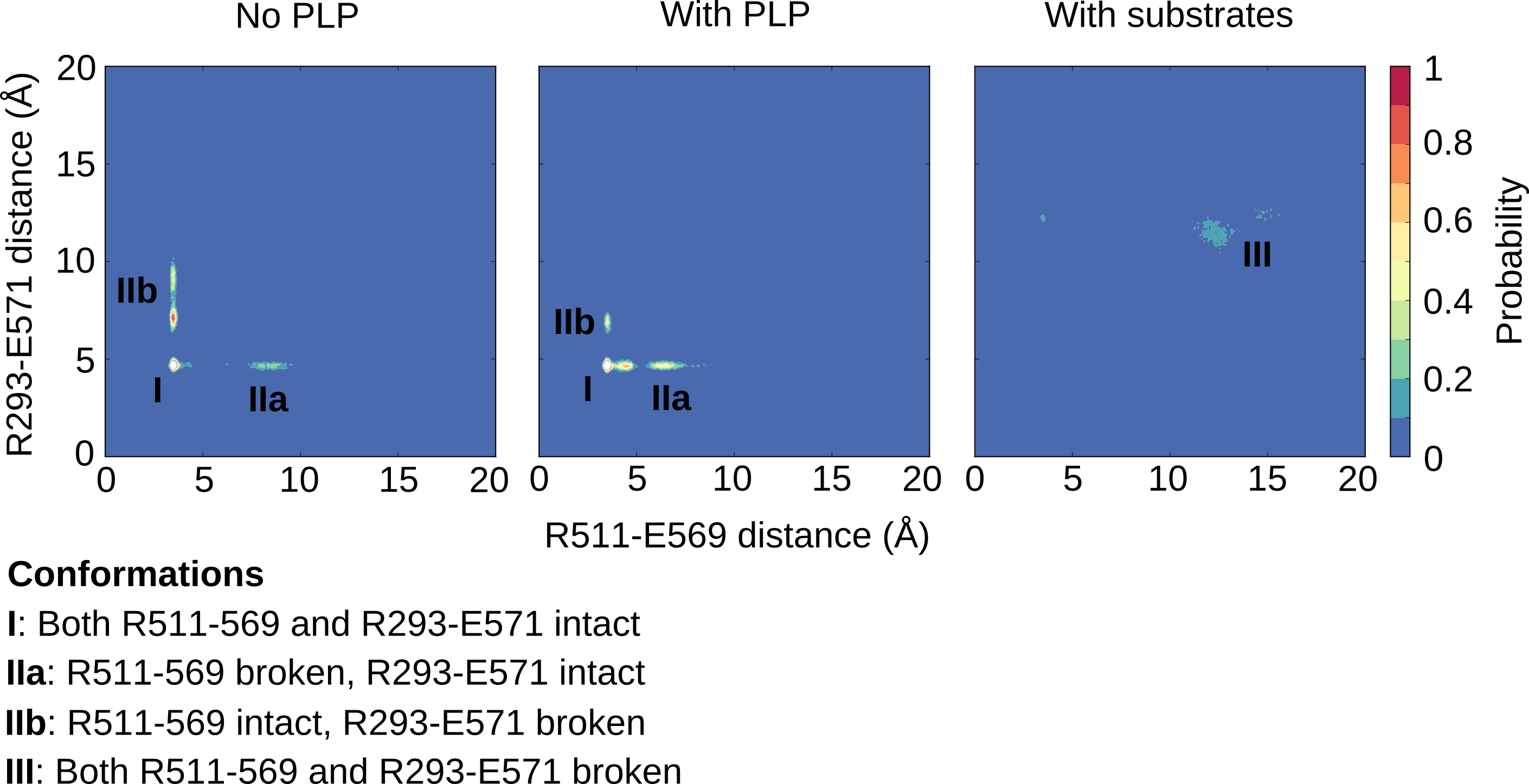


**Supplementary Fig. 9 Conformational landscape of the Ct-extension in terms of salt bridge dynamics.** The figure shows probabilities as a function of R511-E569 distance and R293-E571 distance. To calculate the distance between two residues, the distance between the centers of the guanidium group of arginine and the carboxylate group of glutamate was calculated. Distance values were calculated separately for the two subunits. The data for the two subunits was merged while computing the probabilities. A total of 50 ns of simulation was performed for each of the three systems shown above. Distinct conformations of the Ct-extension have been marked in the figure as “I”, “IIa”, “IIb” and “III”. These conformations have been defined below the probability plots. Source data are provided as a Source Data file.


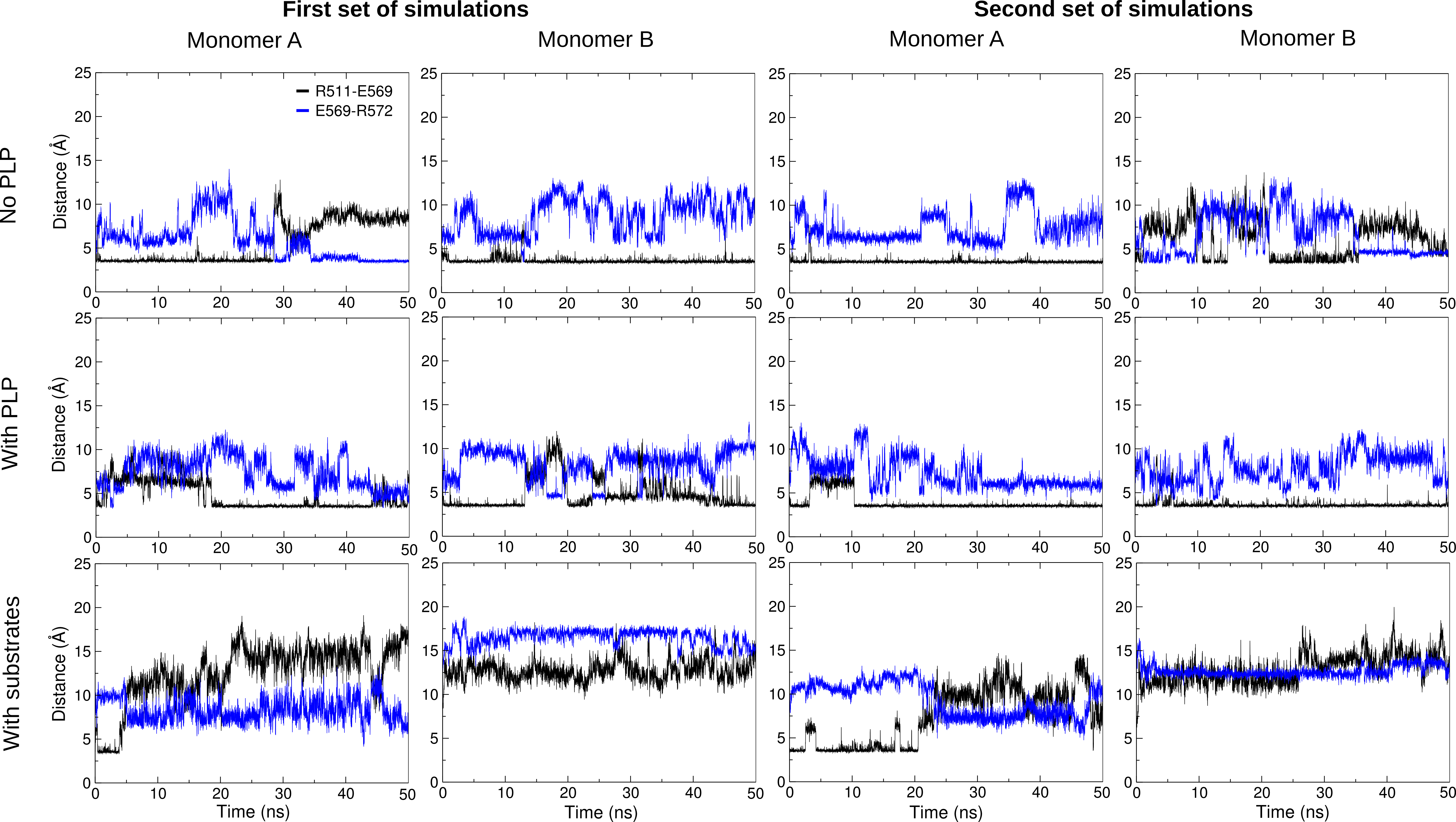


**Supplementary Fig. 10 Dynamics of salt bridges involving E569.** The variation of the distance between the residues participating in salt bridges is shown as a function of simulation time. To calculate the distance between two residues, the center of a given residue was defined as the center of mass of the side-chain guanidinium/amine/carboxylate group, depending on the amino acid type. The distance between the residue centers was then calculated. The salt bridge R511-E569 is stable in the apo form but broken in order to accommodate substrates. E569-R572 is a non-native salt bridge that is observed when R511-E569 is broken. Two sets of simulation were performed and both data are shown here. Source data are provided as a Source Data file.


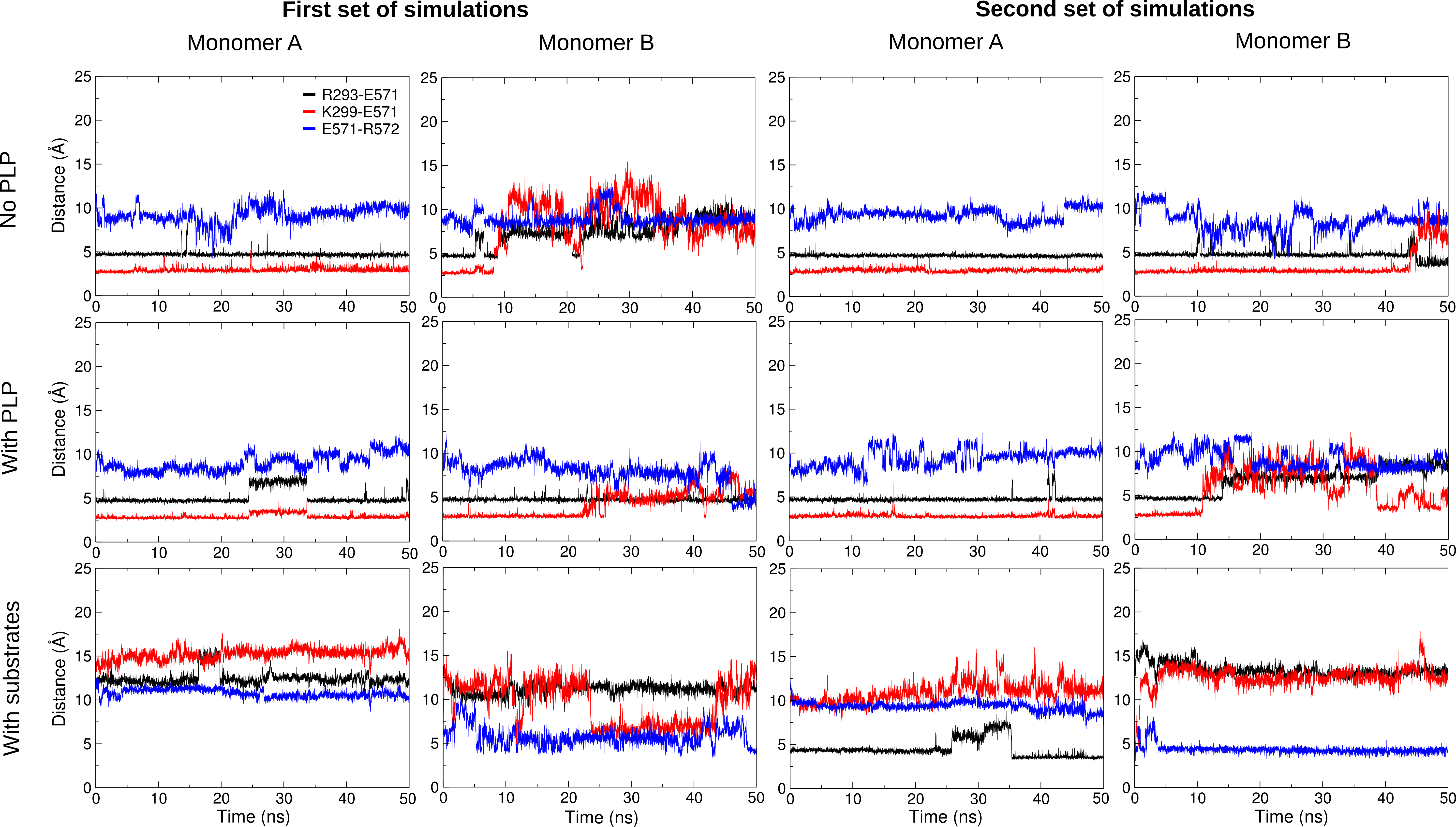


**Supplementary Fig. 11 Dynamics of salt bridges involving E571.** The variation of the distance between the residues participating in salt bridges is shown as a function of simulation time. To calculate the distance between two residues, the center of a given residue was defined as the center of mass of the side-chain guanidinium/amine/carboxylate group, depending on the amino acid type. The distance between residue centers was then calculated. The salt bridges R293-E571 and K299-E571 are broken in order to accommodate substrates. E571-R572 is a non-native salt bridge that is observed when R293-E571 and K299-E571 are broken. Two sets of simulation were performed and both data are shown here. Source data are provided as a Source Data file.


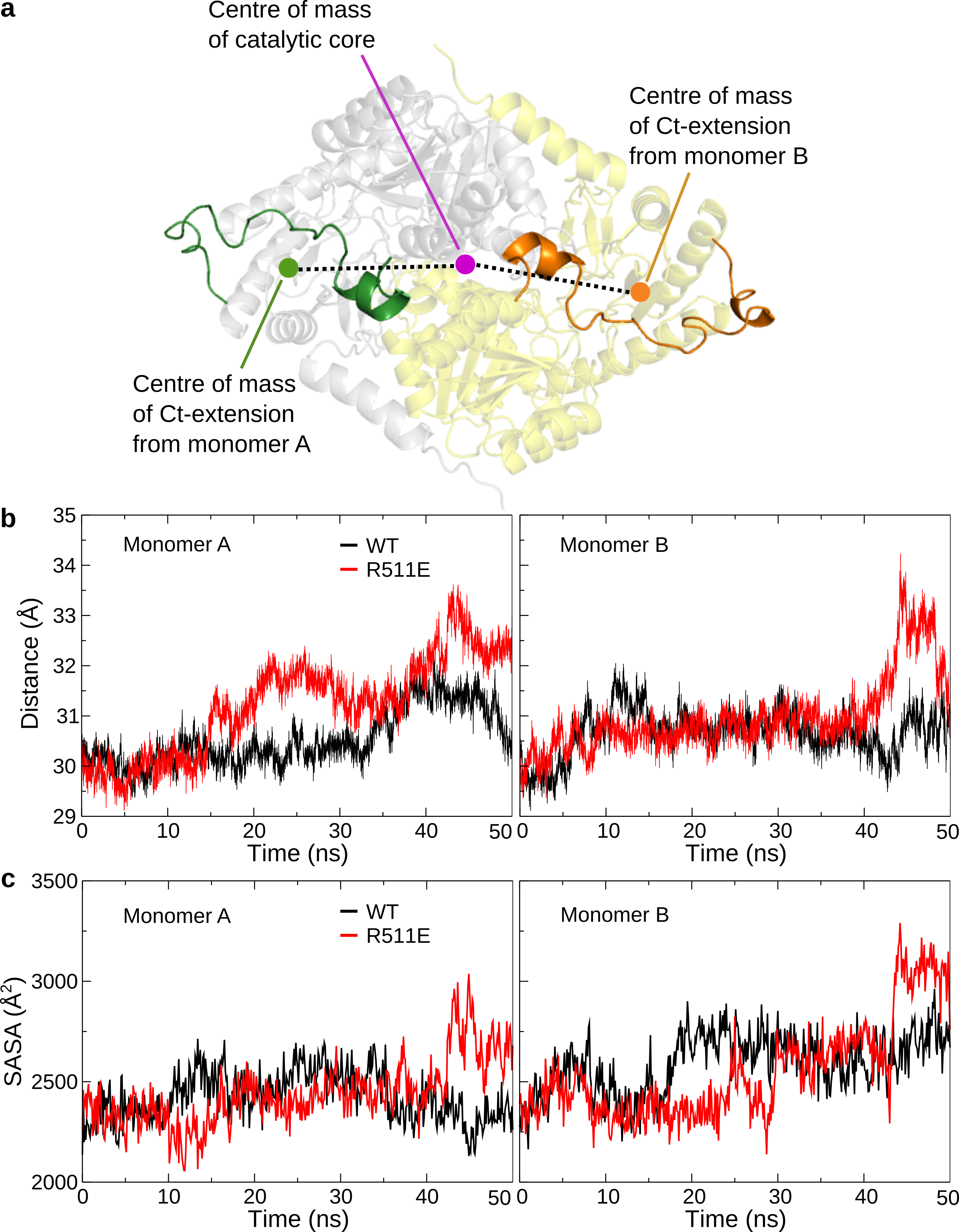


**Supplementary Fig. 12 Accessibility of the Ct-extension.** **(a)** In the current MD simulations, the distance between the centre of mass of the catalytic core of the homodimer (purple dot) and the centre of mass of Ct-extensions from each of the two monomers (orange and green dots) is being measured. These distances are marked by two dotted lines. **(b)** Variation of the distance defined in (a). The R511E model exists in conformations in which the Ct-extension has moved away from the catalytic core, especially in the last 10 ns. **(c)** Variation of solvent accessible surface area (SASA) of the Ct-extension over the course of the simulation. The R511E mutant exists in conformations in which the Ct-extension has moved away from the catalytic core, and therefore has a higher SASA. Source data are provided as a Source Data file.


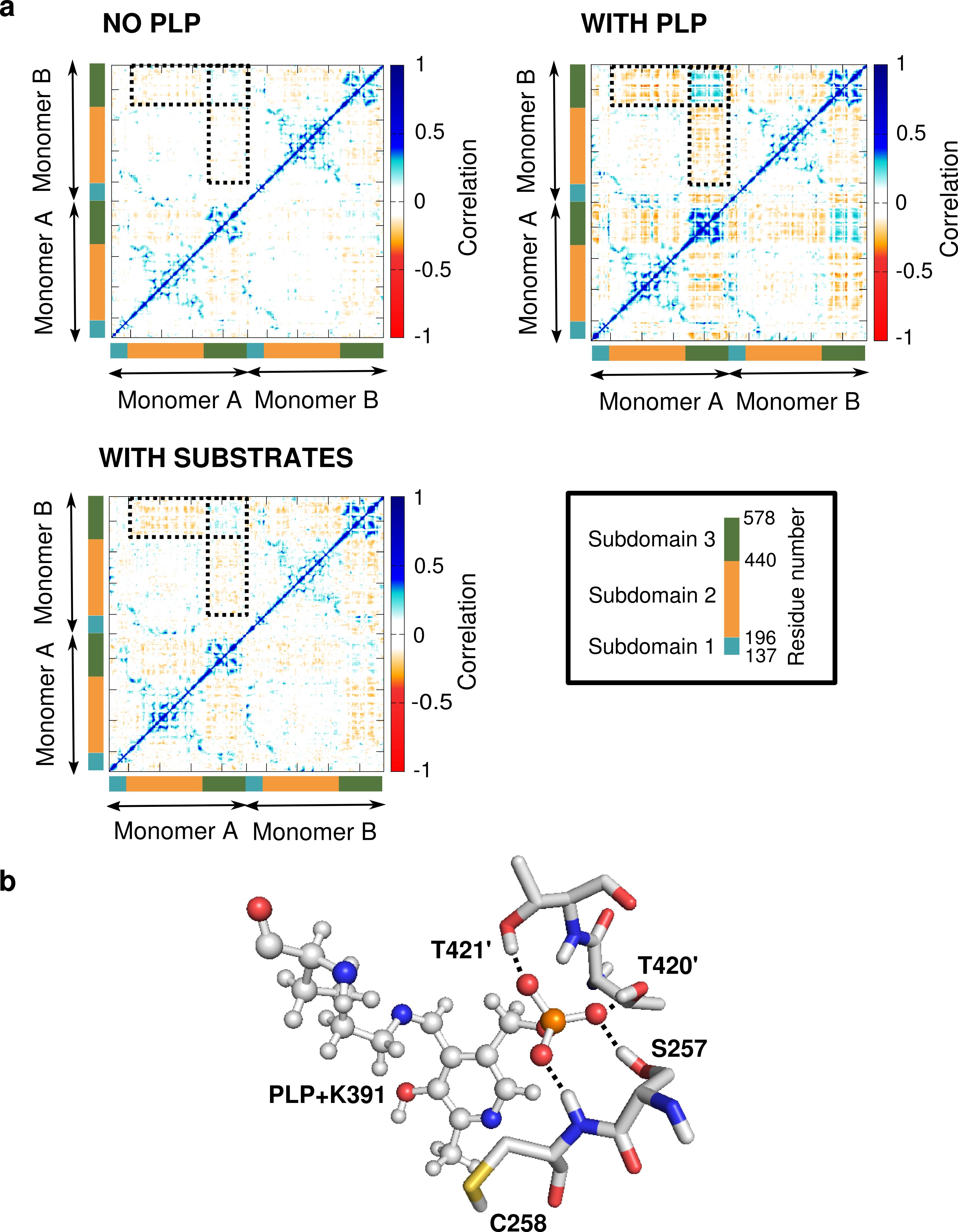


**Supplementary Fig. 13 Dynamic cross correlation maps (DCCM).** (**a**) The correlation in the dynamics of different regions of the protein is shown here. The last 10 ns of the trajectories were considered in the analysis. The dotted rectangles on the plots indicate regions that show increased correlation upon covalent attachment of PLP to the active site. (**b**) Interactions between the phosphate moiety on PLP and surrounding residues. Dotted lines indicate hydrogen bonds. S257 and C258 belong to the same subunit as PLP, and T420’ and T421’ belong to the opposite subunit. Source data for Supplementary Fig. 13a are provided as a Source Data file.


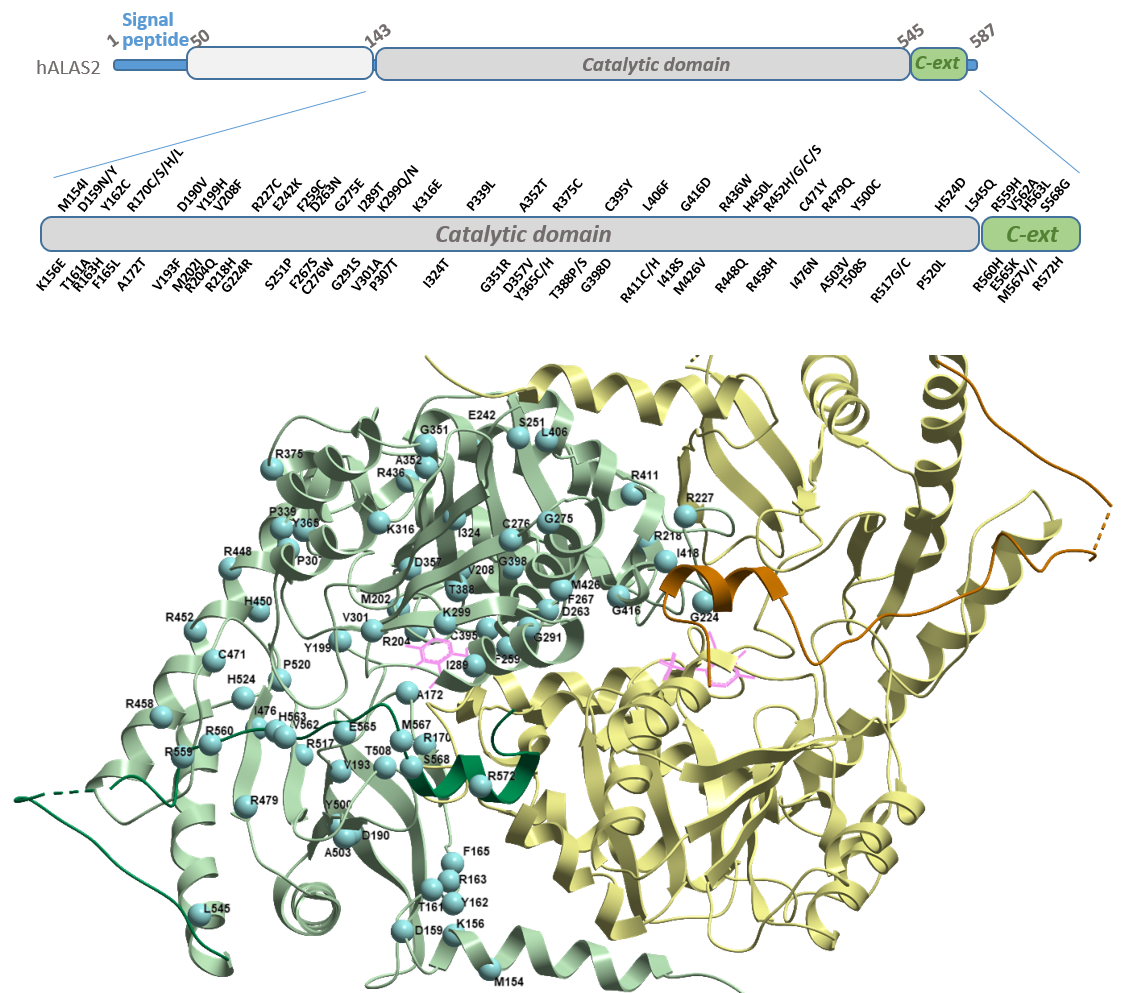


**Supplementary Fig. 14 Mapping of disease-causing missense mutations onto hsALAS2 structure.** Sites of missense mutations that lead to the XLSA disorder (spheres) are mapped onto a domain organisation schematic (*top*) and the hsALAS2 structure shown as a homodimer (green and yellow subunits)(*bottom*).


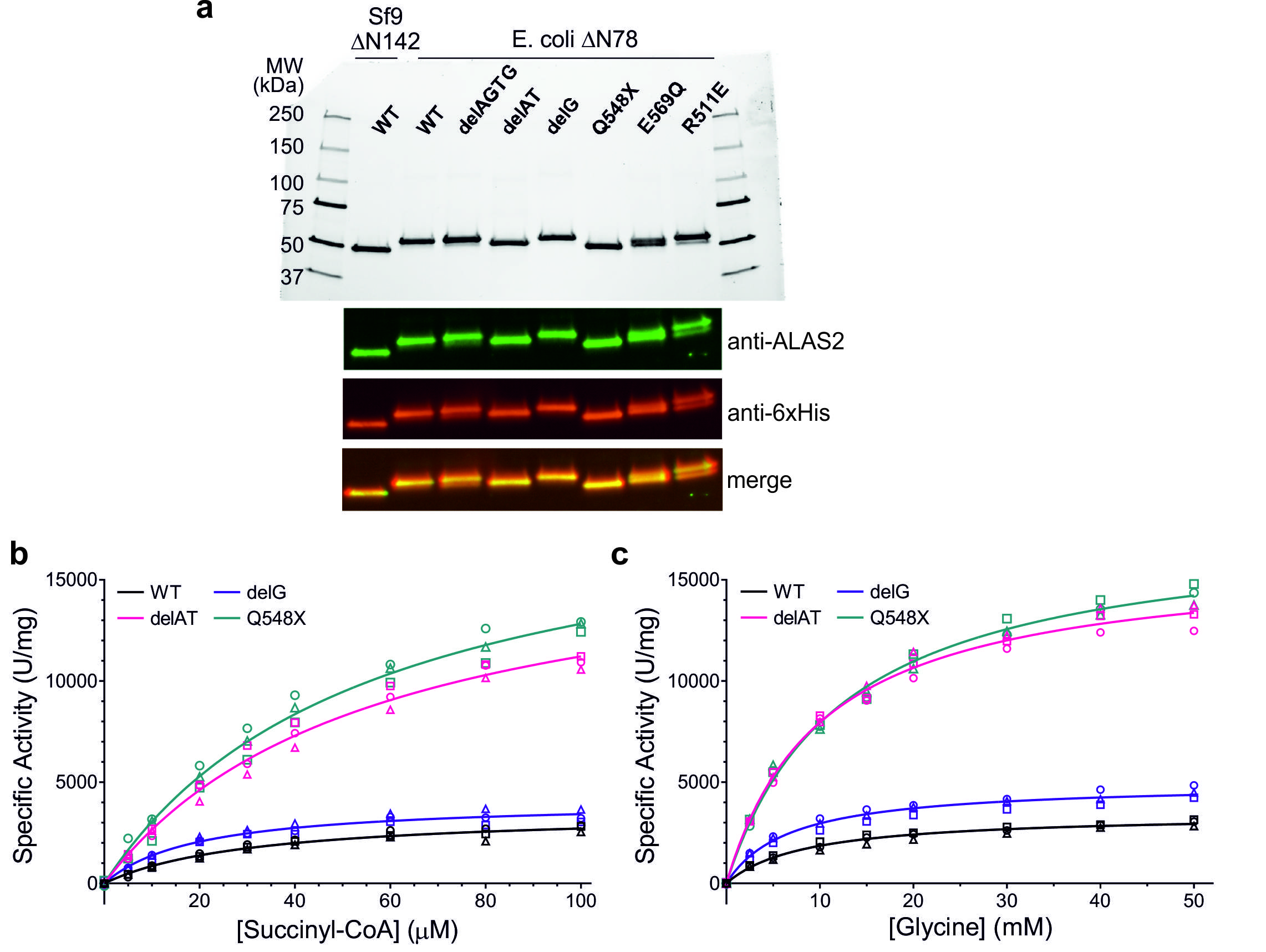


**Supplementary Fig. 15 XLP causing variants of hsALAS2.** (**a**) SDS-PAGE (top) and multiplex western blot (bottom) of recombinant His_6_-tagged hsALAS2 proteins (1 µg/lane and 100 µg/lane, respectively; purity > 90%), expressed in either Sf9 (hsALAS2_ΔN142_) or *E. coli* BL21(DE3) (hsALAS2_ΔN78_) cells as indicated. Results are representative of two independent experiments. Lanes 1 and 10 are molecular weight standards; lane 2 is WT hsALAS2_ΔN142_; lane 3 is WT hsALAS2_ΔN78_; lanes 4-7 are XLP variants reconstructed on hsALAS2_ΔN78_, lanes 8 and 9 are the experimental variants E569Q and R511E reconstructed on hsALAS2_ΔN78_. (**b**,**c**) Michaelis-Menten curves for purified, *E.* coli-expressed hsALAS2_ΔN78_ WT and XLP variants with titration of succinyl-CoA (b) and glycine (c). Regression lines represent the mean of three biological replicates (n=3), with the data for each replicate plotted as distinct symbols (circles, squares and triangles). Specific activity represents initial velocity normalized to protein in each assay (1-4 µg mL^-1^), where succinyl-CoA and glycine titrations were conducted in the presence of 50 mM glycine and 100 µM succinyl-CoA, respectively. Data with statistics are presented in Table 3. Source data are provided as a Source Data file.


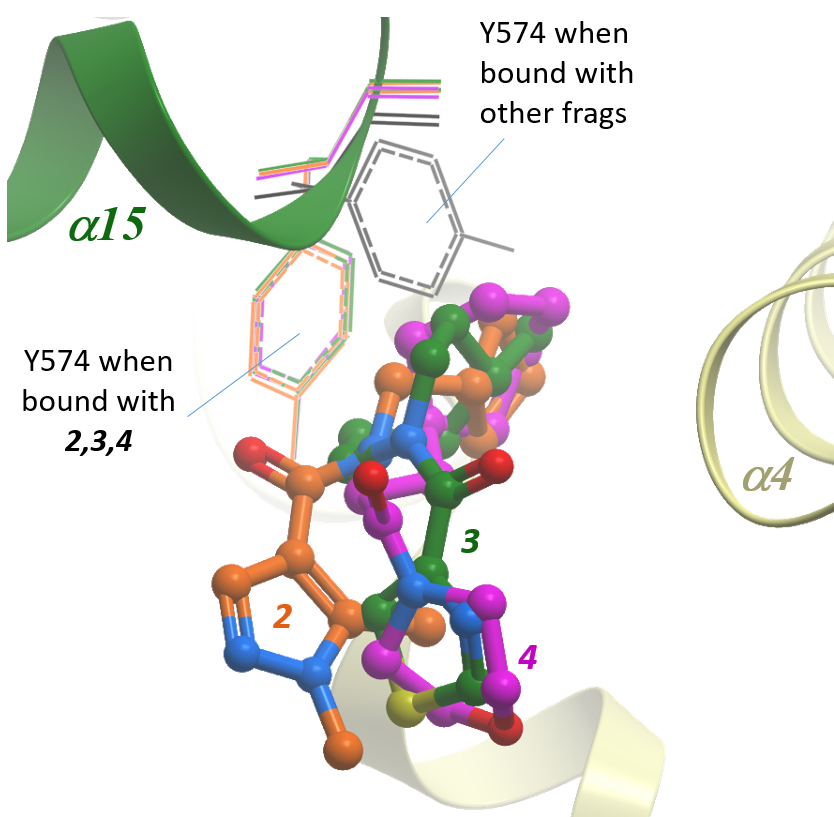


**Supplementary Fig. 16 Alternative conformation of Tyr574.** In structures bound with fragments **2**-**4** (colored sticks), the Tyr574 side-chain (colored lines) adopts a different rotamer conformation from other structures (black line).


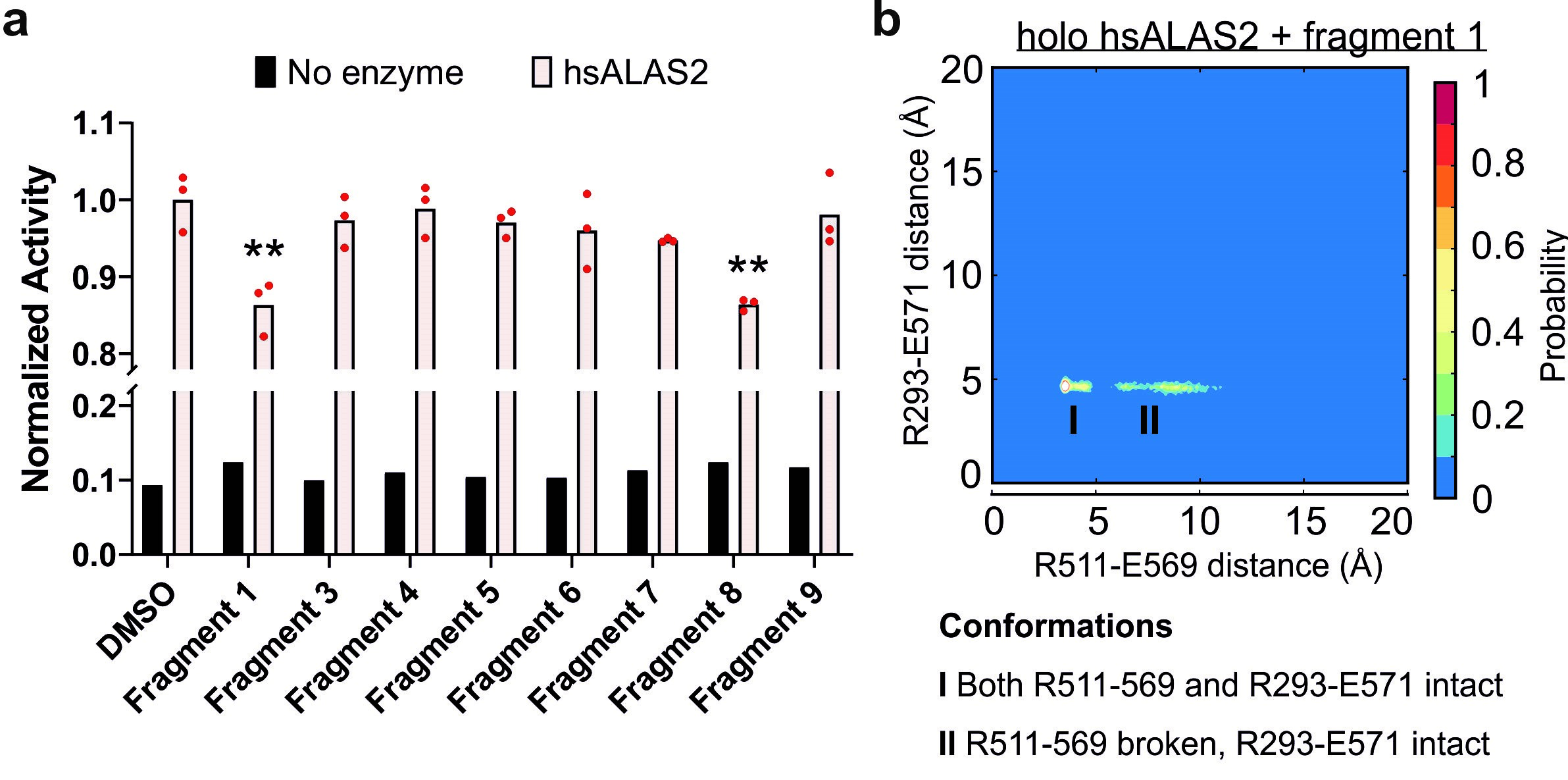


**Supplementary Fig. 17 Impacts of fragment binding on hsALAS2.** (**a**) Enzyme activity of hsALAS2_ΔN78_ expressed in *E. coli* and pretreated with fragments. Purified hsALAS2_ΔN78_ (4 μg mL^-1^) or buffer (no enzyme) was preincubated with fragments **1** and **3-9** at 1 mM concentrations or DMSO (diluent for fragments) at 37°C for 15 min prior to the initiation of reactions as detailed in the Materials and Methods. Assays with protein were run using three separate hsALAS2_ΔN78_ preps (each with n=3 technical replicates). Each plotted activity value (biological replicate) was normalized to protein pretreated with DMSO, and each bar represents the mean of three biological replicates. No-enzyme assays were run in n=3 technical replicates (bars represent the only biological replicate). Statistical significance relative to assays with protein and DMSO was established using one-way ANOVA (*p* = 0.0003) followed by Tukey’s post-hoc analysis (** indicates *p* < 0.01 vs. DMSO control: fragment 1, *p* = 0.0022; fragment 3, *p* = 0.98; fragment 4, *p >* 0.99; fragment 5, *p* = 0.97; fragment 6, *p* = 0.85; fragment 7, *p* = 0.60; fragment 8, *p* = 0.0023; fragment 9, *p >* 0.99). (**b**) Conformational landscape of the Ct-extension in terms of salt bridge dynamics, from MD simulations of *holo* hsALAS2 in the presence of fragment **1**. The figure shows probabilities as a function of R511-E569 distance and R293-E571 distance. The distances have been calculated in a manner similar to that described for fragment-free ALAS2 (Supplementary Fig. 9). Two distinct conformations (I and II) are observed. Only monomer B of the homodimer was included in the analysis, because fragments have dissociated from monomer A during the 50 ns run. Source data are provided as a Source Data file.


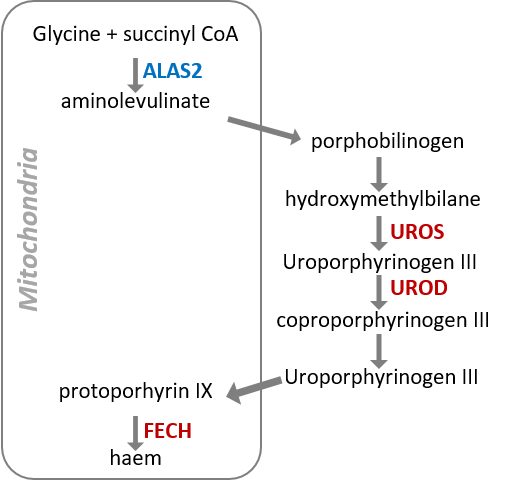


**Supplementary Fig. 18 Pathway of haem biosynthesis in developing erythrocytes.** Enzymes associated with erythropoietic porphyria are labelled in red, and the proposed therapeutic target via the substrate reduction approach, ALAS2, in blue.

**Supplementary References**

1. Astner I, Schulze JO, van den Heuvel J, Jahn D, Schubert WD, Heinz DW. Crystal structure of 5-aminolevulinate synthase, the first enzyme of heme biosynthesis, and its link to XLSA in humans. *EMBO J* **24**, 3166-3177 (2005).

2. Kucerova J, Horvathova M, Mojzikova R, Belohlavkova P, Cermak J, Divoky V. New mutation in erythroid-specific delta-aminolevulinate synthase as the cause of X-linked sideroblastic anemia responsive to pyridoxine. *Acta Haematol* **125**, 193-197 (2011).

3. Cui R, Xu Z, Qin T, Zhang Y, Xiao Z. [Congenital sideroblastic anemia-a new family with identification of K156E mutation of ALAS2 gene and literature review]. *Zhonghua Xue Ye Xue Za Zhi* **35**, 142-146 (2014).

4. Furuyama K*, et al.* Late-onset X-linked sideroblastic anemia following hemodialysis. *Blood* **101**, 4623-4624 (2003).

5. Hurford MT*, et al.* A novel mutation in exon 5 of the ALAS2 gene results in X-linked sideroblastic anemia. *Clin Chim Acta* **321**, 49-53 (2002).

6. Zhu P, Bu D. [A novel mutation of the ALAS2 gene in a family with X-linked sideroblastic anemia]. *Zhonghua Xue Ye Xue Za Zhi* **21**, 478-481 (2000).

7. Moreno-Carralero MI*, et al.* Missense variants in ALAS2 gene in five patients. *Int J Lab Hematol* **41**, e5-e9 (2019).

8. Fujiwara T*, et al.* A novel heterozygous ALAS2 mutation in a female with macrocytic sideroblastic anemia resembling myelodysplastic syndrome with ring sideroblasts: a case report and literature review. *Ann Hematol* **96**, 1955-1957 (2017).

9. Cotter PD, Rucknagel DL, Bishop DF. X-linked sideroblastic anemia: identification of the mutation in the erythroid-specific delta-aminolevulinate synthase gene (ALAS2) in the original family described by Cooley. *Blood* **84**, 3915-3924 (1994).

10. Bergmann AK*, et al.* Systematic molecular genetic analysis of congenital sideroblastic anemia: evidence for genetic heterogeneity and identification of novel mutations. *Pediatr Blood Cancer* **54**, 273-278 (2010).

11. May A, Bishop DF. The molecular biology and pyridoxine responsiveness of X-linked sideroblastic anaemia. *Haematologica* **83**, 56-70 (1998).

12. Yu HJ*, et al.* Mutation Analysis of X-linked Sideroblastic Anemia in a 12-Month-Old Boy by Massively Parallel Sequencing. *Ann Lab Med* **38**, 389-392 (2018).

13. Furuyama K, Sassa S. Multiple mechanisms for hereditary sideroblastic anemia. *Cell Mol Biol (Noisy-le-grand)* **48**, 5-10 (2002).

14. Harigae H, Furuyama K. Hereditary sideroblastic anemia: pathophysiology and gene mutations. *Int J Hematol* **92**, 425-431 (2010).

15. Edgar AJ, Vidyatilake HM, Wickramasinghe SN. X-linked sideroblastic anaemia due to a mutation in the erythroid 5-aminolaevulinate synthase gene leading to an arginine170 to leucine substitution. *Eur J Haematol* **61**, 55-58 (1998).

16. Ducamp S*, et al.* Sideroblastic anemia: molecular analysis of the ALAS2 gene in a series of 29 probands and functional studies of 10 missense mutations. *Hum Mutat* **32**, 590-597 (2011).

17. Cotter PD*, et al.* Late-onset X-linked sideroblastic anemia. Missense mutations in the erythroid delta-aminolevulinate synthase (ALAS2) gene in two pyridoxine-responsive patients initially diagnosed with acquired refractory anemia and ringed sideroblasts. *J Clin Invest* **96**, 2090-2096 (1995).

18. Furuyama K*, et al.* Pyridoxine refractory X-linked sideroblastic anemia caused by a point mutation in the erythroid 5-aminolevulinate synthase gene. *Blood* **90**, 822-830 (1997).

19. Liu G*, et al.* Mutation spectrum in Chinese patients affected by congenital sideroblastic anemia and a search for a genotype-phenotype relationship. *Haematologica* **98**, e158-160 (2013).

20. Cotter PD*, et al.* Four new mutations in the erythroid-specific 5-aminolevulinate synthase (ALAS2) gene causing X-linked sideroblastic anemia: increased pyridoxine responsiveness after removal of iron overload by phlebotomy and coinheritance of hereditary hemochromatosis. *Blood* **93**, 1757-1769 (1999).

21. Harigae H*, et al.* A novel mutation of the erythroid-specific gamma-Aminolevulinate synthase gene in a patient with non-inherited pyridoxine-responsive sideroblastic anemia. *Am J Hematol* **62**, 112-114 (1999).

22. Mendez M, Moreno-Carralero MI, Morado-Arias M, Fernandez-Jimenez MC, de la Iglesia Inigo S, Moran-Jimenez MJ. Sideroblastic anemia: functional study of two novel missense mutations in ALAS2. *Mol Genet Genomic Med* **4**, 273-282 (2016).

23. Rose C*, et al.* Lethal ALAS2 mutation in males X-linked sideroblastic anaemia. *Br J Haematol* **178**, 648-651 (2017).

24. Katsurada T*, et al.* A Japanese family with X-linked sideroblastic anemia affecting females and manifesting as macrocytic anemia. *Int J Hematol* **103**, 713-717 (2016).

25. Le Rouzic MA*, et al.* Non syndromic childhood onset congenital sideroblastic anemia: A report of 13 patients identified with an ALAS2 or SLC25A38 mutation. *Blood Cells Mol Dis* **66**, 11-18 (2017).

26. Rivera CE, Heath AP. Identification of a new mutation in erythroid-specific delta-aminolevulinate synthase in a patient with congential sideroblastic anemia. *Blood* **94(Suppl)**, 19b (1999).

27. Garcon L, Kannengiesser C. A double red cells population in a woman with a microcytic anemia. *Blood* **123**, 808 (2014).

28. Creasey T, Biss T, Lambert J, Smith F, Clark B, Carey P. Pyridoxine-sensitive X-linked 'sideroblastic' anaemia in the absence of ring sideroblasts - molecular diagnosis. *Br J Haematol* **180**, 10 (2018).

29. Bluteau O*, et al.* A landscape of germ line mutations in a cohort of inherited bone marrow failure patients. *Blood* **131**, 717-732 (2018).

30. Bottomley SS, Wise PD, Wasson EG, Carpenter NJ. X-linked sideroblastic anemia in 10 female probands due to ALAS2 mutations and skewed X chromosome inactivation. *Am J Hum Genet* **63 (Suppl)**, A352 (1998).

31. Percy MJ, Cuthbert RJ, May A, McMullin MF. A novel mutation, Ile289Thr, in the ALAS2 gene in a family with pyridoxine responsive sideroblastic anaemia. *J Clin Pathol* **59**, 1002 (2006).

32. Prades E, Chambon C, Dailey TA, Dailey HA, Briere J, Grandchamp B. A new mutation of the ALAS2 gene in a large family with X-linked sideroblastic anemia. *Hum Genet* **95**, 424-428 (1995).

33. Chen C*, et al.* [Genetic diagnosis of a Chinese pedigree with X-Linked sideroblastic anemia: a case report and literature review]. *Zhonghua Xue Ye Xue Za Zhi* **37**, 154-156 (2016).

34. May A, Kerr BC, Whatley SD, Woolf J. The differential diagnosis of inherited sideroblastic anaemia *Haematologica* **91 (s1)**, 24 (2006).

35. Bottomley SS. Sideroblastic anemias. In: *Wintrobe's Clinical Hematology* (ed^(eds Greer JP*, et al.*). 12th edn. Lippincott Williams & Wilkins, Inc (2009).

36. Sankaran VG*, et al.* X-linked macrocytic dyserythropoietic anemia in females with an ALAS2 mutation. *J Clin Invest* **125**, 1665-1669 (2015).

37. Cox TC, Bottomley SS, Wiley JS, Bawden MJ, Matthews CS, May BK. X-linked pyridoxine-responsive sideroblastic anemia due to a Thr388-to-Ser substitution in erythroid 5-aminolevulinate synthase. *N Engl J Med* **330**, 675-679 (1994).

38. Cazzola M, May A, Bergamaschi G, Cerani P, Rosti V, Bishop DF. Familial-skewed X-chromosome inactivation as a predisposing factor for late-onset X-linked sideroblastic anemia in carrier females. *Blood* **96**, 4363-4365 (2000).

39. Rollon N, Fernandez-Jimenez MC, Moreno-Carralero MI, Murga-Fernandez MJ, Moran-Jimenez MJ. Microcytic anemia in a pregnant woman: beyond iron deficiency. *Int J Hematol* **101**, 514-519 (2015).

40. Furuyama K*, et al.* R411C mutation of the ALAS2 gene encodes a pyridoxine-responsive enzyme with low activity. *Br J Haematol* **103**, 839-841 (1998).

41. Goncalves P, Pereira JC, Ribeiro ML. Gene symbol: ALAS2. Disease: sideroblastic anaemia. *Hum Genet* **115**, 532 (2004).

42. Bottomley SS, May BK, Cox TC, Cotter PD, Bishop DF. Molecular defects of erythroid 5-aminolevulinate synthase in X-linked sideroblastic anemia. *J Bioenerg Biomembr* **27**, 161-168 (1995).

43. Moon SY, Jun IJ, Kim JE, Lee SJ, Kim HK, Yoon SS. A novel hemizygous I418S mutation in the ALAS2 gene in a young Korean man with X-linked sideroblastic anemia. *Ann Lab Med* **34**, 159-162 (2014).

44. Aivado M*, et al.* X-linked sideroblastic anemia associated with a novel ALAS2 mutation and unfortunate skewed X-chromosome inactivation patterns. *Blood Cells Mol Dis* **37**, 40-45 (2006).

45. Lee JS, Gu J, Yoo HJ, Koh Y, Kim HK. A Novel ALAS2 Mutation Resulting in Variable Phenotypes and Pyridoxine Response in a Family with X-linked Sideroblastic Anemia. *Ann Clin Lab Sci* **47**, 319-322 (2017).

46. May A, Fitzsimons E. Sideroblastic anaemia. *Baillieres Clin Haematol* **7**, 851-879 (1994).

47. Sussman NL, Lee PL, Dries AM, Schwartz MR, Barton JC. Multi-organ iron overload in an African-American man with ALAS2 R452S and SLC40A1 R561G. *Acta Haematol* **120**, 168-173 (2008).

48. Collins TS, Arcasoy MO. Iron overload due to X-linked sideroblastic anemia in an African American man. *Am J Med* **116**, 501-502 (2004).

49. Lee PL, Reid TJ, 3rd, Bottomley SS, Barton JC. Sideroblastic anemia, iron overload, and ALAS2 R452S in African-American males: phenotype and genotype features of five unrelated patients. *Am J Hematol* **86**, 787-789 (2011).

50. Furuyama K*, et al.* Arg452 substitution of the erythroid-specific 5-aminolaevulinate synthase, a hot spot mutation in X-linked sideroblastic anaemia, does not itself affect enzyme activity. *Eur J Haematol* **76**, 33-41 (2006).

51. Barton JC, Lee PL. Disparate phenotypic expression of ALAS2 R452H (nt 1407 G --> A) in two brothers, one with severe sideroblastic anemia and iron overload, hepatic cirrhosis, and hepatocellular carcinoma. *Blood Cells Mol Dis* **36**, 342-346 (2006).

52. Edgar AJ, Losowsky MS, Noble JS, Wickramasinghe SN. Identification of an arginine452 to histidine substitution in the erythroid 5-aminolaevulinate synthetase gene in a large pedigree with X-linked hereditary sideroblastic anaemia. *Eur J Haematol* **58**, 1-4 (1997).

53. Donker AE*, et al.* X-linked sideroblastic anaemia due to ALAS(2) mutations in the Netherlands: a disease in disguise. *Neth J Med* **72**, 210-217 (2014).

54. Kawakami T*, et al.* [Successful treatment of X-linked sideroblastic anemia with ALAS2 R452H mutation using vitamin B6]. *Rinsho Ketsueki* **59**, 401-406 (2018).

55. Pereira JC, Gutierrez EO, Ribeiro ML. Gene symbol: ALAS2. Disease: sideroblastic anaemia. *Hum Genet* **115**, 533 (2004).

56. An W*, et al.* Mutation analysis of Chinese sporadic congenital sideroblastic anemia by targeted capture sequencing. *J Hematol Oncol* **8**, 55 (2015).

57. Cotter PD, Baumann M, Bishop DF. Enzymatic defect in "X-linked" sideroblastic anemia: molecular evidence for erythroid delta-aminolevulinate synthase deficiency. *Proc Natl Acad Sci U S A* **89**, 4028-4032 (1992).

58. Doshi BS, Abramowsky C, Briones M, Bunting ST. Concomitant a novel ALAS2 mutation and GATA1 mutation in a newborn: a case report and review of the literature. *Am J Blood Res* **4**, 41-45 (2014).

59. Pereira JC, Barbot J, Ribeiro ML. Novel human pathological mutations. Gene symbol: ALAS2. Disease: sideroblastic anaemia. *Hum Genet* **126**, 333 (2009).

60. Aguiar E, Freitas MI, Barbot J. Different haematological picture of congenital sideroblastic anaemia in a hemizygote and a heterozygote. *Br J Haematol* **166**, 469 (2014).

61. Lee PL, Barton JC, Rao SV, Acton RT, Adler BK, Beutler E. Three kinships with ALAS2 P520L (c. 1559 C --> T) mutation, two in association with severe iron overload, and one with sideroblastic anemia and severe iron overload. *Blood Cells Mol Dis* **36**, 292-297 (2006).

62. Edgar AJ, Wickramasinghe SN. Hereditary sideroblastic anaemia due to a mutation in exon 10 of the erythroid 5-aminolaevulinate synthase gene. *Br J Haematol* **100**, 389-392 (1998).

63. Cazzola M, May A, Bergamaschi G, Cerani P, Ferrillo S, Bishop DF. Absent phenotypic expression of X-linked sideroblastic anemia in one of 2 brothers with a novel ALAS2 mutation. *Blood* **100**, 4236-4238 (2002).

64. Bishop DF, Tchaikovskii V, Hoffbrand AV, Fraser ME, Margolis S. X-linked sideroblastic anemia due to carboxyl-terminal ALAS2 mutations that cause loss of binding to the beta-subunit of succinyl-CoA synthetase (SUCLA2). *J Biol Chem* **287**, 28943-28955 (2012).

65. Harigae H*, et al.* A novel mutation of the erythroid-specific delta-aminolaevulinate synthase gene in a patient with X-linked sideroblastic anaemia. *Br J Haematol* **106**, 175-177 (1999).
