# Supplementary material for: Human aminolevulinate synthase structure reveals a eukaryotic-specific autoinhibitory loop regulating substrate binding and product release
Source: Nat Commun. 2020 Jun 4;11:2813. doi: 10.1038/s41467-020-16586-x (PMC7272653; doi:10.1038/s41467-020-16586-x)
Supplement: Supplementary file 3 — Description of Additional Supplementary Files [file 41467_2020_16586_MOESM3_ESM.docx]

**Description of Supplementary Files**

**File Name: Supplementary Movie 1**

**Description:** First principal component. The video shows motions along the first principal component of the enzyme in the presence of substrates. The first principal component captures the most dominant motions of the protein. Monomer A is shown in white and monomer B in yellow. The Ct-extensions of the two monomers are shown in green and orange, respectively. The approximate location of the active site is indicated with the help of the Lys391 residue, which is shown as violet spheres. For obtaining the first principal component, a principal component analysis was performed on a 50 ns trajectory of hsALAS2 in the presence of substrates.

**File Name: Supplementary Movie 2**

**Description:** Second principal component. The video shows motions along the second principal component of the enzyme in the presence of substrates. Monomer A is shown in white and monomer B in yellow. The Ct-extensions of the two monomers are shown in green and orange, respectively. The approximate location of the active site is indicated with the help of the Lys391 residue, which is shown as violet spheres. For obtaining the second principal component, a principal component analysis was performed on a 50 ns trajectory of hsALAS2 in the presence of substrates.

**File Name: Supplementary Sofeware 1**

**Description:** GROMACS codes for MD simulations described in Supplementary Figs. 9, 10, 11, 12b, 13a and 17b.

**File Name: Supplementary Data 1**

**Description:** Data collection and refinement statistics for fragment-bound hsALAS2 structures.
