# Supplementary material for: Human aminolevulinate synthase structure reveals a eukaryotic-specific autoinhibitory loop regulating substrate binding and product release
Source: Nat Commun. 2020 Jun 4;11:2813. doi: 10.1038/s41467-020-16586-x (PMC7272653; doi:10.1038/s41467-020-16586-x)
Supplement: Supplementary file 8 — Reporting Summary [file 41467_2020_16586_MOESM8_ESM.pdf]

nature research

Corresponding author(s): Wyatt Yue, wyatt.yue@sgc.ox.ac.uk

Last updated by author(s): 08-Mar-2020

Reporting Summary

Nature Research wishes to improve the reproducibility of the work that we publish. This form provides structure for consistency and transparency in reporting. For further information on Nature Research policies, see Authors & Referees and the Editorial Policy Checklist .

Statistics

For all statistical analyses, confirm that the following items are present in the figure legend, table legend, main text, or Methods section.

n/a	Confirmed	
☐	☒	The exact sample size (*n*) for each experimental group/condition, given as a discrete number and unit of measurement
☐	☒	A statement on whether measurements were taken from distinct samples or whether the same sample was measured repeatedly
☒	☐	The statistical test(s) used AND whether they are one- or two-sided<br>*Only common tests should be described solely by name; describe more complex techniques in the Methods section.*
☒	☐	A description of all covariates tested
☒	☐	A description of any assumptions or corrections, such as tests of normality and adjustment for multiple comparisons
☐	☒	A full description of the statistical parameters including central tendency (e.g. means) or other basic estimates (e.g. regression coefficient) AND variation (e.g. standard deviation) or associated estimates of uncertainty (e.g. confidence intervals)
☒	☐	For null hypothesis testing, the test statistic (e.g. *F*, *t*, *r*) with confidence intervals, effect sizes, degrees of freedom and *P* value noted<br>*Give P values as exact values whenever suitable.*
☒	☐	For Bayesian analysis, information on the choice of priors and Markov chain Monte Carlo settings
☒	☐	For hierarchical and complex designs, identification of the appropriate level for tests and full reporting of outcomes
☒	☐	Estimates of effect sizes (e.g. Cohen's *d*, Pearson's *r*), indicating how they were calculated

Our web collection on statistics for biologists contains articles on many of the points above.

Software and code

Policy information about availability of computer code

Data collection	Mx3005p RT-PCR machine (Stratagene), DLS beamline i04, i24, 104-1 and B21, CLARIOstar microplate reader (BMG Labtech)
Data analysis	GraphPad Prism, ATSAS, DAMMIF, DAMAVER, CCP4, Phenix, Scatter, COOT, PHASER, STARANISO, REFMAC5, DIMPLE XChemXplorer pipeline, PANDDA, MODELLER, CHARMM-GUI

For manuscripts utilizing custom algorithms or software that are central to the research but not yet described in published literature, software must be made available to editors/reviewers. We strongly encourage code deposition in a community repository (e.g. GitHub). See the Nature Research guidelines for submitting code & software for further information.

Data

Policy information about availability of data

All manuscripts must include a data availability statement. This statement should provide the following information, where applicable:

- Accession codes, unique identifiers, or web links for publicly available datasets
- A list of figures that have associated raw data
- A description of any restrictions on data availability

All coordinates and structure factors were deposited to the Protein Data Bank (accession codes 6HRH, 5QQY, 5QR1, 5QRA, 5QRC, 5QRD, 5QQW, 5QQX, 5QRE, 5QQU). Main data supporting the findings of this study are available within the article and the Supplementary Information file. Raw data for Tables 2-3, Supplementary Table 4, Fig. 3b-e, and Supplementary Figs. 3a-c, 4a-c, 5, 6, 9-12, 13a, 15a-c, 17a-b are available in the Source Data file. Other data are available from the corresponding author upon reasonable request.

Field-specific reporting

Please select the one below that is the best fit for your research. If you are not sure, read the appropriate sections before making your selection.

- [x] Life sciences
- [ ] Behavioural & social sciences
- [ ] Ecological, evolutionary & environmental sciences

For a reference copy of the document with all sections, see nature.com/documents/nr-reporting-summary-flat.pdf

Life sciences study design

All studies must disclose on these points even when the disclosure is negative.

Sample size	For structural studies, the sample size used was determined by sufficient signal in experiments to ensure confidence in conclusions drawn from data. That is, redundant set of images and frames were collected to ensure completeness during diffraction data processing.
Data exclusions	No data excluded.
Replication	All activity assays were carried out in technical/biological replicates as stated in figure legends. Multiple structures of ALAS2 have been determined. X-ray diffraction experiments were carried out such that each dataset was collected from one crystal.
Randomization	Not applicable - no experimental groups were involved.
Blinding	Not applicable - no group allocation was involved.

Reporting for specific materials, systems and methods

We require information from authors about some types of materials, experimental systems and methods used in many studies. Here, indicate whether each material, system or method listed is relevant to your study. If you are not sure if a list item applies to your research, read the appropriate section before selecting a response.

Materials & experimental systems

n/a	Involved in the study	
[ ]	[x]	Antibodies
[ ]	[x]	Eukaryotic cell lines
[x]	[ ]	Palaeontology
[x]	[ ]	Animals and other organisms
[x]	[ ]	Human research participants
[x]	[ ]	Clinical data

Methods

n/a	Involved in the study	
[x]	[ ]	ChIP-seq
[x]	[ ]	Flow cytometry
[x]	[ ]	MRI-based neuroimaging

Antibodies

Antibodies used	Primary antibodies:<br>1) anti-ALAS2 rabbit polyclonal antibody (custom-made, purified antibody from Invitrogen available through Thermo Scientific, catalog no 21553)<br>2) anti-6xHis mouse monoclonal antibody (Proteintech, Cat No. 66005-1-lg)<br>Secondary antibodies:<br>1) donkey anti-rabbit IgG secondary antibody (Invitrogen, Dylight 550, Thermofisher catalog no SA5-10039)<br>2) goat anti-mouse IgG secondary antibody (Invitrogen, DyLight 650, Thermofisher catalog no SA5-10041
Validation	Validation info can be found on manufacturers' web sites

Eukaryotic cell lines

Policy information about cell lines

Cell line source(s)	Sf9 insect cell line from Invitrogen
Authentication	Cell line was obtained from vendor
Mycoplasma contamination	Cell line was purchased from company, and not further tested for mycoplasma contamination

Commonly misidentified lines<br>(See ICLAC register)	No misidentified lines used.
